# A trimeric coiled-coil motif binds bacterial lipopolysaccharides with picomolar affinity

**DOI:** 10.3389/fcimb.2023.1125482

**Published:** 2023-02-16

**Authors:** Daniel Hatlem, Mikkel Christensen, Nina K. Broeker, Per E. Kristiansen, Reidar Lund, Stefanie Barbirz, Dirk Linke

**Affiliations:** ^1^ Institutt for Biovitenskap, Universitetet i Oslo, Oslo, Norway; ^2^ Kjemisk Institutt, Universitetet i Oslo, Oslo, Norway; ^3^ Department Humanmedizin, HMU Health and Medical University, Potsdam, Germany

**Keywords:** endotoxin, coiled coil, lipopolysaccharide (LPS), LAL assay, outer membrane (OM), gram-negative bacteria

## Abstract

α-helical coiled-coils are ubiquitous protein structures in all living organisms. For decades, modified coiled-coils sequences have been used in biotechnology, vaccine development, and biochemical research to induce protein oligomerization, and form self-assembled protein scaffolds. A prominent model for the versatility of coiled-coil sequences is a peptide derived from the yeast transcription factor, GCN4. In this work, we show that its trimeric variant, GCN4-pII, binds bacterial lipopolysaccharides (LPS) from different bacterial species with picomolar affinity. LPS molecules are highly immunogenic, toxic glycolipids that comprise the outer leaflet of the outer membrane of Gram-negative bacteria. Using scattering techniques and electron microscopy, we show how GCN4-pII breaks down LPS micelles in solution. Our findings suggest that the GCN4-pII peptide and derivatives thereof could be used for novel LPS detection and removal solutions with high relevance to the production and quality control of biopharmaceuticals and other biomedical products, where even minuscule amounts of residual LPS can be lethal.

## 1 Introduction

**Figure 1:**
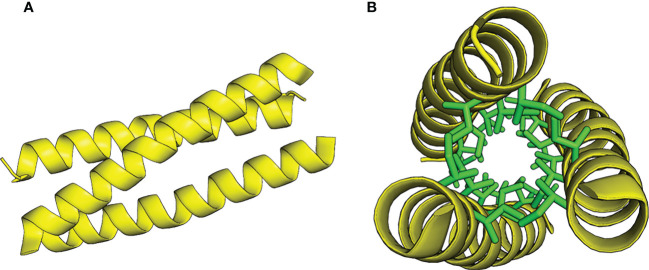
Model of the GCN4-pII coiled-coil trimer structure, adapted from PDB 2YO0 (Hartmann et al., 2012). **(A)** Side view. **(B)** Top view with core isoleucins in a and d positions represented as stick models, colored green.

Coiled-coils are ubiquitous protein elements consisting of two or more amphipathic α-helices wound into supercoiled bundles (Lupas and Gruber, 2005) (**Figure 1**). A characteristic of canonical, amphipathic α-helical coiled-coils is the residual heptad repeat (**a-b-c-d-e-f-g**) where position a and d are occupied by hydrophobic residues, while the others are generally hydrophilic. Straight α-helices have 3.6 residues/turn, and this repeat places the hydrophobic residues on the same face of the helical structure, facilitating the formation of highly stable supercoiled oligomers with the hydrophobic residues facing each other in a hydrophobic core. A prominent and well-studied example of coiled-coil mediated dimerization is the yeast transcription factor GCN4, where the C-terminal domain forms a highly stable dimeric coiled-coil, termed the leucine zipper (GCN4-pLL, where p’**ad**’ refers to the amino acids at position **a** and **d**). Harbury et al. demonstrated that mutating the hydrophobic core residues from leucins to variations of leucins and isoleucins altered the preferred oligomeric state from dimers to trimers (GCN4-pII) and tetramers (GCN4-pLI) (Harbury et al., 1993; Delano and Brünger, 1994). The stability and predictability to which these elements can oligomerize has prompted researchers to use GCN4 zipper variants as chimeric extensions to induce and stabilize protein oligomers. Examples from the past three decades include the dimerization of Tir-kinase (Wang et al., 2002), the stabilization of trimeric autotransporter adhesin wdomains (Alvarez et al., 2008; Hartmann et al., 2012; Muñoz González et al., 2019), and the tetramerization of antibodies (Pack et al., 1995).

**Figure 2:**
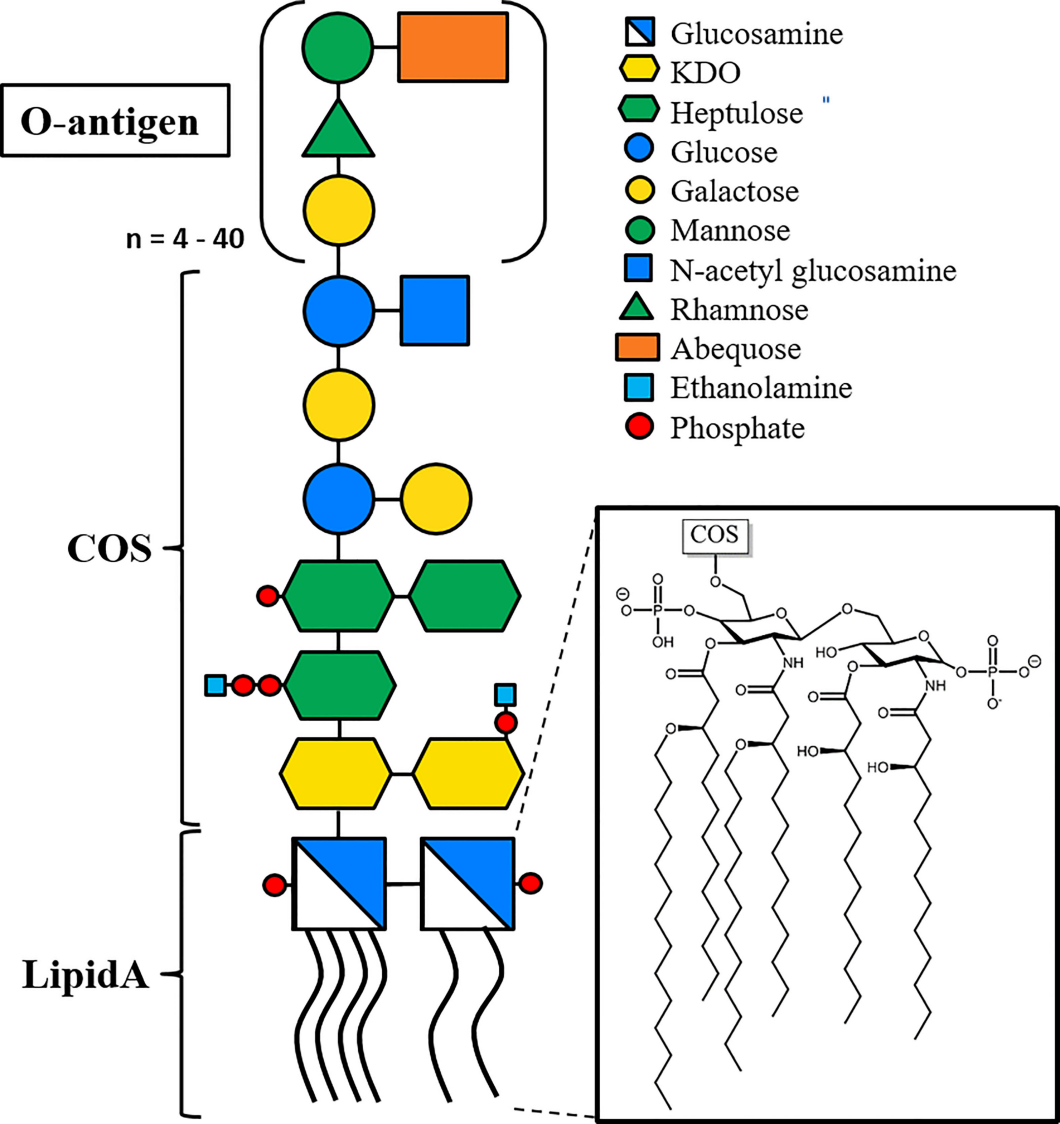
Representative structure of LPS, based on LPS from *Salmonella enterica* sv. Typhimurium. The membrane-embedded lipid A moiety (insert) consists of two phosphoglucosamines with four O-linked and two N-linked acyl chains. The core oligo saccharide (COS) is linked to lipid A *via* a glycosidic bond, and the O-antigen linked to the penultimate COS sugar. The O-antigen consists of a four-sugar repeat varying between 4 and 40 repeat units, with an average of 30 repeats(Peterson and McGroarty, 1985). Lipid A and the two proximal 3-Deoxy-D-manno-oct-2-ulosonic acid (KDO) sugars are highly conserved among Gram-negative species, while the rest of COS and O-antigen are conserved among bacterial species and serotypes, respectively. Glycans are shown using the Consortium for Functional Glycomics (CFG) nomenclature.

Lipopolysaccharides (LPS) constitute the main component in the extracellular leaflet of the Gram-negative outer membrane (Di Lorenzo et al., 2021). They are considered the first line of defense against antimicrobial peptides and hydrophobic toxins such as certain antibiotics (Raetz and Whitfield, 2002). LPS is a glycolipid, composed of a membrane-embedded lipid A moiety, linked to the core oligosaccharide (COS), that in turn is linked to the distal O-antigen polysaccharide (**Figure 2**). The structure of Lipid A and the COS are usually well conserved within Gram-negative species, while the O-antigen is highly variable even between strains and serotypes (Hitchcock et al., 1986). Bacterial strains expressing LPS with the high molecular weight O-antigen form “smooth” type colonies, while knock-out strains lacking the O-antigen form “rough” type colonies, resulting in a smooth/rough terminology to discern LPS with and without O-antigen (Hitchcock et al., 1986). Recognition of lipid A by the human Toll-like receptor 4 results in a strong immune reaction that can result in sepsis and toxic shock syndrome, and doses as low as 5 ng/kg (i.v.) are reported to induce a physiological response in humans, with amounts a low as 1-2 µg being lethal to humans (Sauter and Wolfensberger, 1980; Vaure and Liu, 2014). It is therefore imperative that especially pharmaceutical products are completely LPS-free. Due to the omnipresence of bacteria, and hence, LPS, in the environment, this is difficult to achieve, and requires both highly sensitive tests and challenging LPS removal efforts (Magalhães and Pessoa, 2010). Current methods for LPS removal include for example affinity chromatography with Polymyxin B-based resins that specifically bind LPS; the gold standard for LPS detection today still is the *Limulus* amoebocyte lysate (LAL) assay that relies on the blood of an endangered species, the horseshoe crab (Schneier et al., 2020).

In this work, we show that the trimeric GCN4-pII peptide binds to the conserved lipid A moiety of LPS with an unprecedented affinity, and that GCN4-pII dissolves LPS aggregates in solution. We propose that the extreme affinity between the peptide and the highly conserved lipid A moiety of LPS make the peptide a useful biotechnological tool to bind, remove or detect LPS. Furthermore, we present a proof-of-concept that GCN4-pII can be used to detect LPS with similar sensitivity as the leading commercial alternative, as well as qualitative evidence that GCN4-pII binds LPS isolated from a broad range of Gram-negative species.

## 2 Materials and methods

### 2.1 Expression and purification of proteins

*Salmonella* adhesin A (SadA) His-tagged constructs K3-His, K9-His ad K14-His flanked by GCN4 adaptors were produced as described earlier (Alvarez et al., 2008; Hartmann et al., 2012) (see **Figure S1** for overview of the constructs used in this study). Transformed BL21-Gold (DE3) were grown in 2 L ZYP-5052 medium (Studier, 2005), and overexpression induced at OD_600_ = 0.6 by adding anhydrotetracycline (AHTC) to final concentration of 200 ng/mL, followed by expression overnight at 30 ˚C. The cells were pelleted by centrifugation at 6000 × *g* (Beckman JLA 8.1000 rotor) for 30 minutes and resuspended in 20 mL Tris/HCl pH 7.4, 40 mM NaCl, 5 mM MgCl_2_ containing 200 µL EDTA-free Protease Inhibitor Cocktail (Merck) and DNase I (New England Biolabs). The resuspended cells were lysed using a French press, and the resulting lysate was diluted in 50 mL equilibration buffer (20 mM Tris/HCl pH 7.9, 5 M guanidine hydrochloride, 0.5 M NaCl, 10% glycerol) and incubated at room temperature for 1 hour while stirring, followed by centrifugation at 75 000 × *g* (Beckman Ti 70 rotor) for 1 hour to remove any undissolved particulates. The resulting solution containing the denatured protein was loaded onto a 20 mL Ni Sepharose Excel column (GE life sciences) pre-equilibrated with equilibration buffer. Following application of the sample, the column was washed with 4 column volumes equilibration buffer and eluted using a 0-100% gradient elution buffer (20 mM Tris/HCl, pH 7.5, 5 M guanidine hydrochloride, 0.5 M NaCl, 10% glycerol, 500 mM imidazole). The eluted fractions were analyzed by SDS-PAGE, and fractions containing the protein of interest was pooled and refolded by dialyzing twice against 2 L refolding buffer (20 mM MOPS pH 7.4, 350 mM NaCl, 10% glycerol) over night.

### 2.2 LPS production and purification

LPS from all species except *Bartonella henselae* was produced by inoculating 6 × 1 L cultures in 2 L baffled flasks in lysogeny broth (LB) from a 20 mL preculture (originating from a single bacterial colony, see **Table S1** for strains used). The cultures were grown over night at 37 ˚C in a shaker at 200 rpm. The bacteria were harvested by centrifugation at 6000 × *g* (Beckman JLA 8.1000 rotor) for 30 minutes. Further purification followed two different methods depending on the type of LPS. *B. henselae* was grown in *Bartonella* liquid media as previously described (Riess et al., 2008). LPS from *Porphyromonas gingivalis* was acquired from a commercial source (*In vivo*Gen).

Rough LPS from *Salmonella enterica ssp. enterica serovar* Typhimurium (referred to as *S.* typhimurium from here on) WaaL and WaaC knock-out strains, *Bartonella henselae*, and *Neisseria lactamica* was purified as described by Galanos et al. (Galanos et al., 1969), using phenol-chloroform-petroleum ether extraction. Following harvest, the bacterial pellet was washed 3 times with 40 mL ethanol and once with acetone, and left overnight under an airflow. The dried pellet was homogenized using a mortar and pestle and dissolved in a 40 ml mixture of 90% (W/V) phenol in milliQ-water (18.2 MΩ·cm at 25°C, MQ), chloroform, and petroleum ether in a ratio of 2:5:8. After one hour incubation on a shaker, the undissolved material was pelleted at 4200 × *g* for 15 minutes and the supernatant collected. Chloroform and petroleum ether was removed under an airflow for 4 hours or until the phenol started crystallizing. The solution was resuspended by heating to 40 ˚C, and MQ added dropwise (3 × 5 drops) under stirring until the LPS precipitated. The LPS was pelleted at 4200 × *g* for 15 minutes, and more water added to the supernatant to collect any residual LPS. The pellets were washed two times with 10 mL 80% (W/V) phenol, and taken up in 20 mL MQ before centrifugation at 100 000 × *g* (Beckman, MLA-50 rotor) for one hour. The final pellet was taken up in 50 mL MQ and lyophilized to yield pure LPS.

Smooth LPS from *S.* typhimurium wt and *Vibrio cholerae* was purified as described by Darveau et al. (Darveau and Hancock, 1983). The bacteria were washed twice and resuspended in 40 mL 10 mM Tris-HCl pH 8.0, 2 mM MgCl_2_, and lysed with a French press, followed by additional disruption by sonication. The resulting suspension was incubated with 200 µg/mL DNase I, 50 µg/mL RNase A (New England Biolabs) overnight while stirring at 37 ˚C. 14 mL 0.5 M EDTA in 10 mM Tris-HCL pH 8.0, 7 mL 20% SDS in 10 mM Tris-HCl pH 8.0, and 7 mL 10 mM Tris-HCl pH 8.0 were added to the suspension, and the LPS micelles were further disrupted by sonication. The solution was centrifuged at 39 000 × *g* (Sorvall SS-34 rotor) for 30 minutes at 20 ˚C to pellet undissolved cell components, the supernatant was frozen and lyophilized. The lyophilized crude extract was dissolved in 40 mL MQ, and the LPS was precipitated with 2 volumes of ice-cold ethanol and 0.375 M MgCl_2_ at -40 ˚C overnight. The precipitated LPS was centrifuged at 11 000 × *g* (Sorvall SLA-3000 rotor) for 15 minutes at 4 ˚C, and the resulting pellet was resuspended in the same volume of 90% (W/V) phenol at 65 ˚C for 30 min while stirring. The mixture was centrifuged at 4000 × *g* for 10 min to accelerate phase separation. The water phase was collected, and the phenol phase extracted once more with MQ. The water phases were pooled, and phenol was extracted using ¼ the volume of chloroform. The water phase was placed under an airflow overnight to evaporate any residual organic solvent, and dialyzed against 5 L MQ for 3 days using a 500 MWCO dialysis membrane (SpectraPor^®^). The dialyzed LPS was frozen and lyophilized. The lyophilized LPS was dissolved in 10 mM Tris-HCl, 2 mM MgCl_2_ pH 8 and incubated for 1 h at 37°C with 10 µg/mL DNase I and 10 µg/mL RNase A. Afterwards the remaining protein was digested by 15 µg/mL proteinase K at 56°C for 3 h. In a last step the LPS was ultracentrifuged at 250 000 × *g* for 2 hours at 15 ˚C (Sorvall T-865 rotor). The pellet was resuspended in MQ, ultracentrifuged a second time, before final resuspension in MQ, and lyophilized to yield pure LPS. The purity of the isolated LPS was controlled by tricine-SDS-polyacrylamide gel electrophoresis and UV-vis spectroscopy (Marolda et al., 2006).

### 2.3 Preparation of O-antigen polysaccharides

O-antigen polysaccharides were isolated from wild type *S.* typhimurium LPS by mild acid hydrolyzation of the glycosidic bond connecting lipid A to the proximal KDO sugar (Raetz and Whitfield, 2002). 4-5 mg/mL *S.* typhimurium LPS was dissolved in 10% acetic acid and incubated at 100 ˚C for 1 hour. The resulting lipid A was removed from the solution by centrifugation at 10 000 × *g* for 30 minutes at 4 ˚C. The supernatant containing the polysaccharide was frozen and lyophilized overnight. This protocol results in a polysaccharide with one KDO sugar at the reducing end of the polysaccharide.

### 2.4 ELISA-like tailspike adsorption assay

The ELITA assay was first described by Schmidt et al. (Schmidt et al., 2016) using whole bacteria. Here, we modified the assay for use with purified proteins in a Nunc 96-flat-well MaxiSorp plate (see **Figure S2** for details on the experimental setup). The wells were saturated by incubating with 100 µl 10µg/mL of either K9-His or K14-His in PBS buffer overnight at 4°C. Following a 2 hour blocking step with 2% bovine serum albumin (BSA) in PBS, 100 µL dilutions of *S.* typhimurium LPS ranging from 200 µg/mL to 0.0023 µg/mL were added as a binding partner and incubated for 1 hour. To detect the fraction of GCN4-bound LPS after removal of the solution,100 µL 10 µg/mL P22 tailspike protein (P22TSP) (Schmidt et al., 2016) with an N-terminal Strep-tag^®^II (IBA) was added, and incubated for one hour, before the wells were finally incubated with 100 µL 1:10 000 StrepTactin-conjugated horseradish peroxidase (IBA, Göttingen) for one hour. The assay was developed with 2,2’-azino-bis 3-ethylbenzothiazoline-6-sulphonic acid (ABTS, Sigma-Aldrich) for 30-60 min and read at 407 nm using a plate reader. The wells were washed 3 times with 150 µL PBS buffer containing 0.1% BSA between each of the above steps (Tween-20 was omitted for these experiments since it interfered with the assay). The average background signal (0 µg/mL LPS) was subtracted from each average signal, and propagation of error calculated by adding the individual standard deviations for the triplicates to the baseline in quadrature. The dose-response curve and dissociation constant K_D_ was calculated by fitting the data to the Hill equation as follows:


$$Y=\;\frac{Y_{max}{\left[L\right]}^{n}}{{\left(K_{D}\right)}^{n}+\;{\left[L\right]}^{n}}$$

where Y denotes the fraction of occupied receptor binding sites, Y_max_ the maximal binding, [L] the concentration of free ligand, and n the number of binding sites. The average molecular weight of smooth *S.* typhimurium LPS was calculated to 22 kDa assuming an average of 30 O-antigen repeats per polysaccharide (Peterson and McGroarty, 1985; Raetz and Whitfield, 2002; Schmidt et al., 2016). Kinetic and statistical parameters can be found in **Table S4**.

### 2.5 ELISA-like biotinylated LPS assay

Black 96-well Greiner or Nunc Maxisorp microplates were coated by incubating 100 µl 10 µg/mL SadA K9 in PBS-buffer overnight at 4 ˚C. Wells were blocked the next day by incubating 150 µL 2% BSA in PBS for 2 hours at room temperature. 4 replicates of 100 µL dilutions of biotinylated *E. coli* 0111:B4 LPS (*In vivo*gen) ranging from 0.06 ng/mL to 1 ng/mL were added as a binding partner and incubated for 1 hour. The plate was washed 3 times with 150 µL PBS + 0.1% BSA, incubated with 100 µL 1:10 000 StrepTactin-conjugated horse radish peroxidase (IBA) for one hour, and developed with QuantaRed fluorescent substrate (Thermo) for 15 min. Fluorescence was measured at excitation: 550 nm, emission: 610 nm. Each dilution was subsequently tested with the LAL-assay (Pierce™) to compare the sensitivities.

### 2.6 Surface plasmon resonance experiments

**Figure 3:**
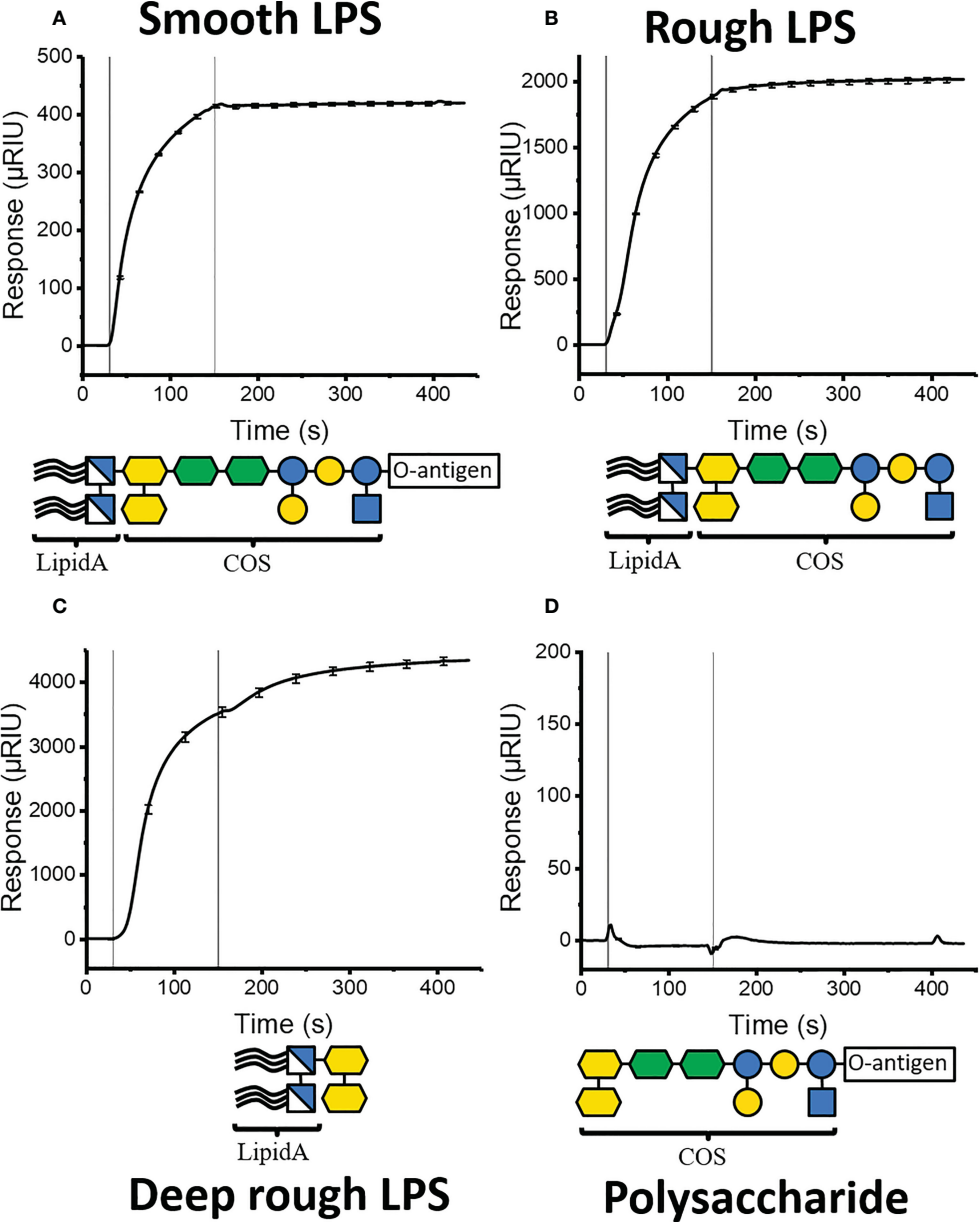
SPR binding isotherms following injection of different *S.* typhimurium LPS components to immobilized K9-GCN4. Vertical lines indicate the start and end of injection. **(A)** injection of whole LPS **(B)** Injection of rough LPS, lacking O-antigen. **(C)** Injection of deep rough LPS, lacking all sugars except the two proximal KDOs. **(D)** Injection of LPS polysaccharide lacking lipid A.

Initial SPR-experiments (**Figure 3**) were conducted on a Reichert 2SPR system at ambient temperature using PBS-E (PBS pH 7.4 + 5 mM EDTA) running buffer. The proteins were diluted to 50 µg/mL in 20 mM sodium acetate buffer pH 4.5 and immobilized to a CMD200 sensor chip (Xantec Bioanalytics, Düsseldorf, Germany) using NHS-EDC amine coupling (Fischer, 2010) to a response of 2000 – 9000 µRIU. Following a comparison of different reference compounds (ethanolamine, BSA, casein, and skimmed milk) (Péterfi et al., 2000), ethanolamine was chosen as the standard coating for the reference channel for all experiments. Later SPR experiments were recorded on an OpenSPR (Nicoya) instrument using a carboxyl sensor chip following the same coupling protocol and conditions.

All LPS ligands were solubilized to 1 mg/mL in running buffer by extrusion (21 passes through a 100 µm filter at 70 ˚C). The experiments were performed at 50 µL/min flowrate in triplicates. Each sample was injected over both measurement and reference channel for 90 s (Reichert) and 150 s (Nicoya) followed by 300 s dissociation. The chip was regenerated by 2 × 30 s injection of regeneration buffer (0.05% (w/w) CHAPS, 0.05% (w/w) Zwittergent 3-12, 0.05% (v/v) Tween 80, 0.05% (v/v) Tween 20, and 0.05% (v/v) Triton X-100) (Andersson et al., 1999). The measurement data was exported to TraceDrawer (RidgeView instruments lab) for processing, and final curves generated using Origin (OriginLab corporation). The signal for each construct was normalized to K9 using the following formula $S=S_{0}\;\left(\frac{\frac{R}{MW}}{\frac{R_{K9}}{MW_{K9}}}\right)$ where S is the normalized signal, S_0_ is the original signal, R is the response following immobilization, and MW is the molecular weight of the construct. All isotherms can be found in **Figures S4–S6**.

### 2.7 Electron microscopy

*S.* typhimurium rough LPS in PBS buffer was extruded (21 passes through 100 nm at 70° C). 45 nM (0.15 mg/mL) LPS was imaged alone and together with 140 nM (0.136 mg/mL) synthetic GCN4-pII peptide (1:3 LPS : GCN4-pII molar ratio). The samples were prepared in 10 µl drops on parafilm. Carbon coated (300 mesh) copper grids were floated on these drops for ten minutes to ensure adhesion, followed by 5 × 1 minute washing with filtered H2O. Next, the samples were passed through a drop of filtered 1% uranyl acetate (UA), followed by 60 seconds incubation on a 1% UA drop and 5 × 1 min washing steps, as before. Samples were then passed through a drop of filtered 0.4% UA/1.8% methylcellulose, immediately followed by a 2-min incubation on a drop of 0.4% UA/1.8% methylcellulose, followed by pick up in 3.2-mm loops and air-drying. Imaging was performed using a JEOL JEM 1400 120 kV TEM; digital images of the sample were obtained using a TWIPS camera and JEOL software.

### 2.8 Small angle X-ray scattering

SAXS experiments on mixtures of LPS and GCN4-PII peptides were conducted at beamline P12 at the Deutsches Elektronen-Synchrotron (DESY) in Hamburg, Germany. The data were collected using an energy of 10.0 keV and a detector distance of 2.953 m covering a *Q*-range ( $Q=\;\frac{4\pi sin\left(\theta /2\right)}{\lambda }$ , where Θ is the scattering angle, and λ is the X-ray wavelength) of 0.0029 Å^-1^< *Q*< 0.73 Å^-1^. The data was calibrated to an absolute intensity scale using water as a primary standard. The pair correlation function were calculated using the GNOM/ATSAS software package (Franke et al., 2017) and the model fits were performed using the QtiKWS software (Pipich, V., QtiKWS. 2019).

### 2.9 Theoretical scattering models of SAXS data

The scattering curves from mixtures of LPS and GCN4-pII peptides were fitted on an absolute scale using models that allow for short cylindrical micelles, flexible worm-like micelles, free trimeric cylinder bundles, and large aggregates respectively. As the typical radius of an α-helix is too small to be resolved by SAXS(Cochran et al., 1952), the trimeric peptide bundle was modeled as three parallel, solid cylinders using the form factor given by Oster and Riley (Oster and Riley, 1952). The micellar LPS structure was modelled using a combination of well-established form factors for cylindrical and worm-like core-shell micelles (Pedersen et al., 1995; Pedersen, 2008; Jensen et al., 2014). The peptide was allowed to enter the micelles in the hydrophobic core and/or shell region, allowing us to quantify both the amount and the rough location of the peptide in the micelles. The peptide location was determined by the fit parameters *f_ps_* and *f_pc_* =1- *f_ps_*, being the fraction of peptide bound to the shell (polysaccharide part) and the core (lipid part), respectively. The fraction of peptide bound to the micelles were determined by the fit parameter *f_pb_*, while the unbound peptide, given by 1– *f_pb_*, was assumed to exist as free, trimeric peptide bundles in the solution. Furthermore, larger aggregates were modeled using classical aggregate scattering (Debye and Bueche, 1949), and a term describing so-called blob scattering were added to account for significant local short-range correlations of the molecules in the presence of water which influences scattering at high *Q* (Debye, 1947). The combination of these models, results in the following expression for scattering intensity of the system:


$$I\left(Q\right)=I_{cyl}\left(Q\right)f_{sc}+\left(1-f_{sc}\right)I_{worms}\left(Q\right)+\left(1-f_{bp}\right)I_{pep,free}\left(Q\right)+I_{cluster}\left(Q\right)+I_{blob}\left(Q\right)$$

where *I_sc_* (*Q*), *I_worms_* (*Q*), *I_pep,free_* (*Q*), *I_blob_* (*Q*) and *I_cluster_* (*Q*) describe the scattering intensities of the cylindrical micelles, worm-like micelles, free peptide bundles, clusters and blob scattering, respectively. As the model is complex and involves many fit parameters which can lead to overfitting, the parameters describing the scattering from the peptide bundles were first determined from a sample of pure peptide and kept fixed in the fits of the mixed samples.

### 2.10 Scattering models

The model fitting was performed using combinations of well-established core-shell models (Pedersen et al., 1995; Pedersen, 2008; Jensen et al., 2014) on an absolute scale using molecular constraints and known concentrations of the system. The contrasts for the LPS and peptide molecules were obtained from the scattering lengths densities *ρ_i_* of the tail group (lipid A) and head group (polysaccharides) of LPS, the GCN4-pII peptide and the solvent. The scattering length densities were calculated as $\rho_{i}=\sum_{i=1}^{N}b_{i}/V_{i}$ with *b_i_* being the scattering length and *V_i_* the volume of the moiety. For X-rays, the scattering lengths are calculated as *b_i_ =Z_i_r_e_* where *Z_i_* is the number of electrons and *r_e_* is the Thomson radius. Molecular volumes for the tail and head groups of LPS were calculated based on mass densities found to fit for SAXS curves of pure LPS (*d_tail_* and *d_head_*), which corresponds well with values similar to known densities of other lipids and oligosaccharides (Clifton et al., 2015). Small changes in the density of the tail group of LPS, however, were needed to describe the scattering curves from the mixtures. The relative uncertainties in the fit parameters caused by these variations were thus estimated by finding the range of densities that could be used to successfully describe the scattering curve of pure LPS.

To account for the introduction of peptide molecules in the LPS micelles, pseudo particles with scattering length densities *ρ _pseudo,tail_* and *ρ _pseudo,head_*, and volumes *V _pseudo,tail_* and *V_pseudo, head_* were introduced. These were calculated and normalized using known volume fractions of LPS, the fraction of peptide bound to LPS micelles and the fraction of bound peptide located in the micelle core and shell, respectively. The peptide location was determined by the fit parameter *f_ps_* for the fraction of peptide in the shell, and correspondingly *f_pc_ =1– f_ps_* for the core. Lastly, the fraction of bound peptide was determined by the fit parameter *f_pb_*. The remaining peptide, given by *1– f_pb_*, was assumed to exist as free trimeric peptide bundles in the solution with dimensions determined by fits of pure peptide in solution.

### 2.11 Form factors for short cylindrical micelles and worm-like micelles with elliptical cross-sections

Short, cylindrical core-shell micelles with elliptical cross-sections can be described with the aspect ratio *ϵ*, core radius *R_core_*, shell thickness *t_shell_* and core length *L_core_*, yielding the minor axis *R_tot_ =R_core_ +t_shell_* and major axis *R_tot_ =ϵR_core_ +t_shell_*. Such cylinders are known to have the orientationally averaged form factor (Rice, 1956):


$$\begin{array}{l} P_{cs,\;cyl}\left(Q\right)=\int_{0}^{\frac{\pi }{2}}\int_{0}^{\frac{\pi }{2}}[\text{Δ}\rho_{shell}V_{tot}\frac{2J_{1}\left(QR_{tot}\left(\phi \right)\sin\alpha \right)}{QR_{tot}\left(\phi \right)\sin\alpha }\cdot \frac{\sin\left(Q(L_{core}+2t_{shell})\cos\frac{\alpha }{2}\right)}{Q(L_{core}+2t_{shell})\cos\frac{\alpha }{2}}\;\\ {+\;\left(\text{Δ}\rho_{core}-\text{Δ}\rho_{shell}\right)V_{core}\frac{2J_{1}\left(QR_{core}\left(\phi \right)\sin\alpha \right)}{QR_{core}\left(\phi \right)\sin\alpha }\cdot \frac{\sin\left(Qt_{shell}\cos\frac{\alpha }{2}\right)}{Qt_{shell}\cos\frac{\alpha }{2}}]}^{2}\sin\alpha \;d\alpha \;d\phi \end{array}$$

where $R_{i}\left(\phi \right)=\sqrt{R_{i}^{2}sin^{2}\left(\phi \right)+{\left(R_{i}\epsilon \right)}^{2}cos^{2}\left(\phi \right)}$ , *J_1_ (x)* is the first order Bessel function of first kind, and where Δ*ρ_shell_*, *V_shell_*, Δ*ρ_core_* and *V_core_* are the scattering contrasts and volumes for the shell and core, respectively

Very long and flexible cylindrical core-shell micelles require a different form factor which includes the so-called Kuhn length, *b*, describing the flexibility of the cylinder. It is only for a contour length of the worm *L_worm_ >b* that these cylinders are flexible and considered to be worm-like. The form factor for such worm-like micelles is given by:


$$\;\;\;P_{cs,\;worm}\left(Q\right)={\left[\text{Δ}\rho_{shell}V_{tot}A_{ell}\left(QR_{tot}(\phi \right))+\left(\text{Δ}\rho_{core}-\text{Δ}\rho_{shell}\right)V_{core}A_{ell}\left(QR_{core}\left(\phi \right)\right)\right]}^{2}P_{chain}\left(Q,L_{worm},b\right)$$

Where *A_ell_* is the scattering amplitude for an elliptical cross-section and is given as


$$\begin{array}{c} A_{ell}\left(x\right)=\int_{0}^{\frac{\pi }{2}}{\left[\frac{2J_{1}x}{x}\right]}^{2}d\phi . \end{array}$$

In the longitudinal direction, the scattering is given by the form factor *P_chain_ (Q, L_worm_, b).* It has been shown by analyzing scattering curves from worm-like chains (Pedersen and Schurtenberger, 1996; Pedersen et al., 1996; Arleth et al., 2002) that the expression can be written as a combination of the contributions from an infinitely thin rod with length *L* (Neugebauer, 1943), and from a random-walk self-avoiding chain contour with length *L_worm_* and Kuhn length *b* (Debye, 1947; Pedersen and Schurtenberger, 1996). This means that the function follows the scattering from the rod at high *Q*, and that of a flexible chain at low *Q*.

### 2.12 Scattering model for trimeric α-helical peptide bundles

Since *α*-helices are too small to be resolved in high detail by small-angle scattering, they are modeled as short, solid cylinders (Cochran et al., 1952; Oster and Riley, 1952). The orientationally averaged form factor for such a cylinder is given as


$$P_{cyl}\left(Q\right)=\text{Δ}\rho^{2}V_{cyl}\int_{0}^{\frac{\pi }{2}}{\left[\frac{2J_{1}\left(QR\sin\alpha \right)}{QR\sin\alpha }\cdot \frac{\sin\left(QL\cos\frac{\alpha }{2}\right)}{QL\cos\frac{\alpha }{2}}\right]}^{2}\sin\alpha d\alpha$$

Where *V_cyl_*, *L* and *R* are the volume, length and radius of the cylinder, respectively. To account for the interaction between the helices, a structure factor *S(q)* is included. For a trimeric bundle, the structure factor can be modeled using the expression (Oster and Riley, 1952)


$$S_{3}\left(q\right)=\frac{1}{9}\left(3+6J_{0}\left(2qRf\right)\right)$$

where *f* is a swelling parameter controlling the distance between the cylinders. One can also account for thermal fluctuations and intrinsic displacements and structural defects by assuming a Gaussian distribution with the width *σ_d_* which yields the following modified structure factor (Förster et al., 2005):


$$S_{tot}\left(q\right)=1+\left(3S_{3}\left(q\right)-1\right)\exp\left(-q^{2}\sigma_{d}^{2}\right)$$

The final differential scattering cross section for can then be given as:


$$I_{pep}\left(Q\right)=n_{pep}P_{cyl}\left(q\right)S_{tot}\left(q\right)$$

where *n_pep_* is the number density of the peptide molecules.

### 2.13 Fitting of pure GCN4-pII

The scattering curve from a sample containing 1.22 mg/mL GCN4-pII was fitted using the trimeric, peptide bundle model derived from PDB-ID: 2YO0 (Hartmann et al., 2012). From the fits we obtained a cylinder radius of 2.4 Å, and a length of 63 +/10- Å. The dimension of a single helical peptide (~5 Å) is similar to what is seen in known crystallographic structures of the peptide. For the length which can be determined with less precision due to the concomitant scattering of larger aggregates at low Q, is slightly longer than expected from the unit cell of the crystal.

### 2.14 Full scattering model

By combining the three presented models, we get a final expression for the total scattering intensity:


$$I\left(Q\right)=I_{cyl}\left(Q\right)f_{sc}+\left(1-f_{sc}\right)I_{worms}\left(Q\right)+\left(1-f_{bp}\right)I_{pep,free}\left(Q\right)+I_{cluster}\left(Q\right)+I_{blob}\left(Q\right)$$

Here, the scattering intensity *I_cluster_* accounts for scattering of large clusters and can be described by the Debye-Bueche like expression(Debye and Bueche, 1949):


$$I_{cluster}\left(Q\right)=\frac{C}{{\left(1+Q^{2}\xi^{2}\right)}^{\alpha }}$$

where *C* is a scaling factor, *ξ* is the cluster size and *α* is an exponent, and *I_blob_ (Q)* describes the so-called blob scattering which is based on the form factor of random-walk Gaussian chains given by Debye (Debye, 1947), and accounts for the significant local short-range correlations of the molecules in the presence of water.

A full list of the fit parameters obtained from the fits can be found in **Table S2**, in addition to a plot of the cross-section dimensions of the micelles as a function of molar ratio in **Figure S3** and **Table S3**.

### 2.15 Limulus amebocyte lysate assay

LAL-assays (Pierce, Thermo Fisher) were conducted following the manufacturers protocol, using the provided *E. coli* (011:B4) Endotoxin Standard for the standard curve. The LPS masking effect of GCN4-pII was measured by spiking 0.25 EU/mL Endotoxin standard to 0.1 and 1 µg/mL GCN4-pII compared to 0.25 EU/mL LPS alone. The signal of 0.1 and 1 µg/mL GCN4-pII was measured alone as control.

### 2.16 Circular dichroism

Spectra were recorded using a Jasco J-810 spectropolarimeter (Jasco International Co) with 0.1 cm path length quartz cuvette. Each sample was scanned five times in the range of 190 to 250 nm with a scanning rate of 50 nm/min at a bandwith of 0.5 nm. Spectra were recorded with an LPS to GCN4-pII ratio of 1:1, in 10 mM Tris pH 7.4 at 37°C using 0.15 mg/mL (41 nM) GCN4-pII and 0.045 mg/mL (13.7 nM) rough *S.* typhimurium LPS. 0.045 mg/mL LPS was measured as background.

### 2.17 Nuclear magnetic resonance spectroscopy

NMR experiments were carried out in Bel-Art™ SP Scienceware™ 5mm O.D. Thin Walled Precision NMR Tubes containing 450 µL 1.5 mM synthetic F_Met_-GCN4-PII peptide (Genscript) in 50 mM NaCl, 7% D_2_O, and 0.2 mM 4,4-dimethyl-4-silapentane-sulfonic acid (DSS). Spectra were acquired at 308 K on a Bruker Avance II 600 MHz NMR spectrometer equipped with a 5 mm ^1^H/^13^C/^15^N-cryoprobe. DSS was used as internal chemical shift standard, and ^13^C and ^15^N was referenced using frequency ratios as described (Wishart et al., 1995). The following spectra were recorded: ^1^H-^1^H TOCSY using a mixing time of 60 and 80 ms (Cavanagh et al., 2007). Spectra were processed using Topspin 4.0 and peaks assigned using CARA 1.9.1 (Keller, 2004).

## 3 Results

### 3.1 GCN4-pII binds to the lipid A moiety of LPS

Based on unpublished observations from an earlier project, we originally intended to investigate a putative interaction between LPS and two domains belonging to the *Salmonella* adhesin A (SadA) (Raghunathan et al., 2011). SadA belongs to a family of fibrous surface proteins, the trimeric autotransporter adhesins, that are composed of trimeric coiled coils, interspaced by small globular domains (Linke et al., 2006). For surface plasmon resonance (SPR) binding analyses, we used two previously described SadA constructs (Alvarez et al., 2008; Hartmann et al., 2012), K9 and K14, containing the putative LPS binding domains, that are stabilized in trimeric form by flanking GCN4-pII segments. As a control, we used SadA K3, a construct that contains only a coiled-coil segment of SadA flanked by GCN4-pII (**Figure S1**). The SadA constructs (K9, K14, and K3) were covalently linked to an SPR-chip to probe binding of different types of LPS fragments (see **Table S1** for an overview of the LPS types used).

Unexpectedly, all three constructs bound LPS, indicating that their only common features, the flanking GCN4-pII segments, were responsible for the previously observed LPS interaction. Injection of smooth LPS immediately gives a response, which approaches a steady state towards the end of injection (**Figure 3A**). During the following buffer injection phase, the signal remains at the plateau, indicating that there is no dissociation of the LPS from the surface. Injection of rough and deep-rough LPS variants (**Figures 3B, C**), shows similar binding curves, except for a slight increase in signal during the dissociation phase. A polysaccharide lacking the lipid A part shows no observable binding (**Figure 3D**).

All injected LPS variants containing the lipid A moiety bind strongly, as opposed to the pure polysaccharide, thus suggesting that the lipid A moiety promotes the observed interaction. However, the low dissociation rate, sample heterogeneity, and the propensity of LPS to form micelles in solution (Sasaki and White, 2008; Richter et al., 2011) impedes a quantitative description of the LPS-GCN4 complex formation. We believe that the increase in signal following injection of the rough and deep-rough variants is inversely proportional to the number of sugar residues in each variant. Particularly, deep-rough LPS has a significantly higher hydrophobic to hydrophilic ratio, adopting a larger, less fluid morphology compared to LPS with longer sugar moieties (Richter et al., 2011). We therefore interpret the signal increase following injection to result from a slower reorganization (and eventually a breakdown) of the deep-rough micelles compared to the smooth variant. Additionally, the sugars have a more similar density to the buffer compared to lipid A, meaning that they contribute less to the total signal. These factors together may explain why the total signal at the end of injection was not proportional to the molar concentrations calculated for each LPS variant.

Our constructs were purified using a 6 × His-tag, which has been implicated to have an endotoxin-depleting effect during purification due to unspecific binding (Mack et al., 2014). To exclude that the His-tag is responsible for the observed binding of LPS, we compared two GCN4-pII flanked SadA constructs identical except for the His-tag (K3, and K3-His). This yielded almost identical curves to each other and to the previous constructs, showing that the His-tag has no measurable effect on the observed affinity (**Figure S6**). Anspach et al. reported that the endotoxin depleting effect of the His-tag only occurs below the isoelectric point of the imidazole side group (pH< 6.0), which is in line with our observations (Anspach, 2001).

Given that GCN4 binds lipid A, we asked ourselves whether the nature of the observed interaction was hydrophobic, electrostatic, or a combination of both. The choice of regeneration solution helps us answer this. Multiple regeneration conditions were screened prior to the experiments (Andersson et al., 1999), and only a mixture of non-denaturing detergents tallying to 0.3% proved effective, indicating a significant hydrophobic contribution to the binding mechanism.

**Figure 4:**
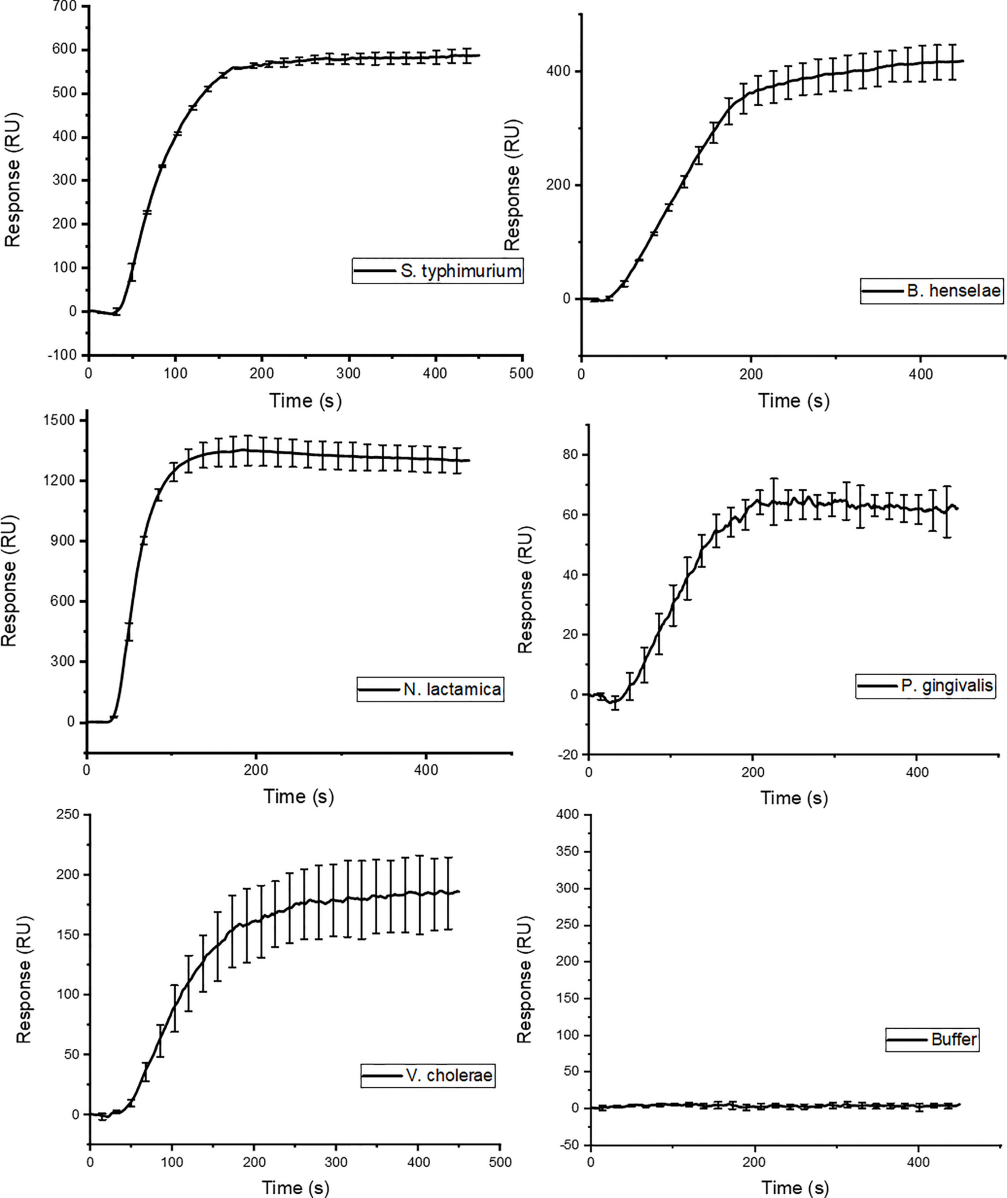
SPR binding isotherm following injection of LPS isolated from different Gram-negative species. The binding curves show the same binding characteristics as the ones observed for the *S.* typhimurium variants in **Figure 3**.

Lipid A is the most conserved part of LPS. We therefore investigated whether GCN4-pII could bind to LPS isolated from a broad range of Gram-negative bacteria with larger variations in both saccharide and lipid A structures. Injection of the different LPS isolates from *B. henselae*, *N. lactamica*, *P. gingivalis*, and *V. cholera* results in similar binding curves to what was observed for *S.* typhimurium (**Figure 4**), with an absent dissociation rate. The different LPS isolates cover a large range of sizes and saccharide compositions, which explains the large differences in signal at plateau.

### 3.2 GCN4-pII binds LPS with an extremely high affinity

**Figure 5:**
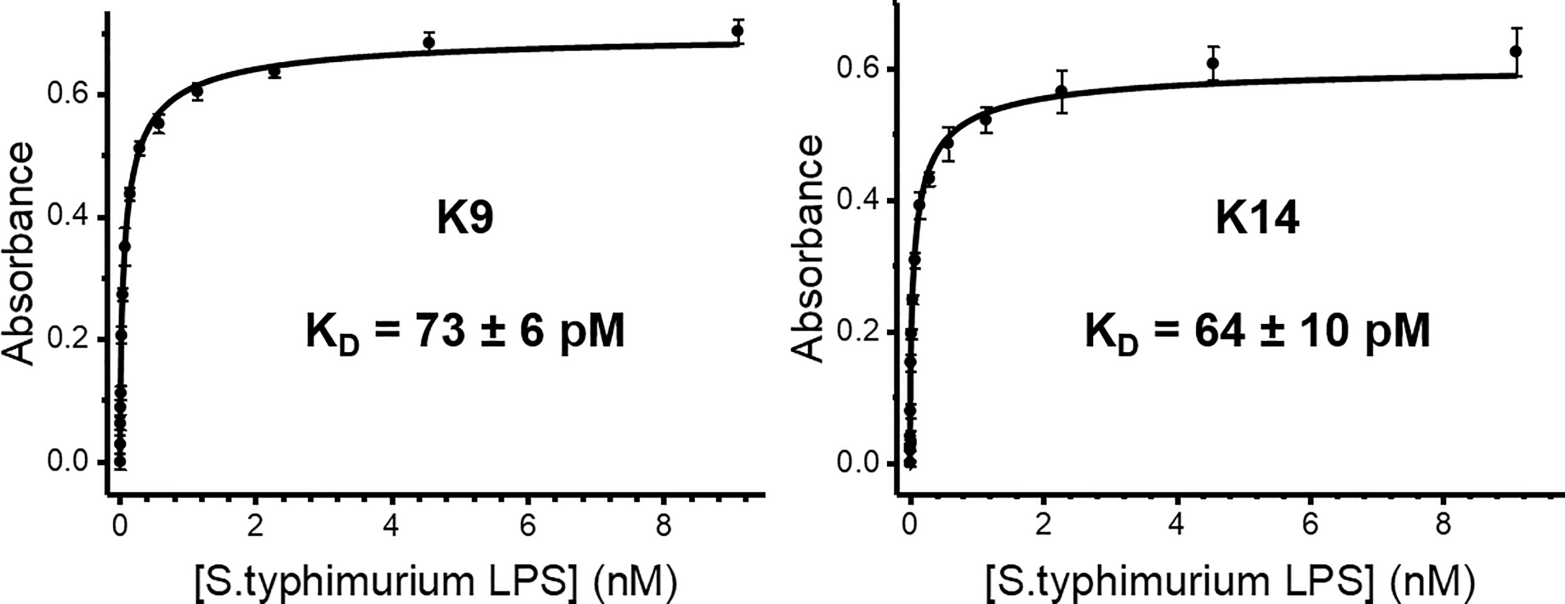
ELITA binding curves of LPS to the two GCN4-containing constructs K9-His and K14-His, shown with K_D,app_ values.

The high affinity observed with SPR did not allow us to quantify the binding affinities of the GCN4-pII/LPS interaction from kinetic rates, mainly because there was no observable dissociation rate. We therefore chose to use a microtiter plate-based assay format for quantification. We immobilized the GCN4-pII constructs in the wells and incubated them with different concentrations of LPS. Bound LPS was then detected using a variant of the ELISA-like tailspike adsorption (ELITA) assay (Schmidt et al., 2016). The assay is similar to a traditional ELISA, except that the primary antibody is replaced with a phage tailspike protein that specifically recognizes the O-antigen of *Salmonella* LPS (**Figure S2**). This setup proved advantageous for our system since it allowed us to use LPS concentrations below the reported critical micelle concentration (CMC) of smooth LPS (Yu et al., 2006; Sasaki and White, 2008). We therefore assume that the LPS molecules exist predominantly as monomers in solution, and not as aggregates, which would have complicated our model. In order to quantify the binding affinity, we attempted to use the Langmuir isotherm for formation of an ideal ligand monolayer on an adsorbing surface as a model to describe the system, as well as the extended Freundlich model, which takes multilayer adsorption into account (Lombardo and Thielemans, 2019). However, these resulted in unsatisfactory curve fits, indicating that the models were not able to explain our data fully. We ultimately used the Hill equation to describe the system. The Hill coefficients of 0.66 and 0.69 (**Table S4**) indicate that there is negative cooperativity between multiple binding sites, which could be explained by steric hindrance caused by the large size of the LPS ligand and the trimeric nature of the protein. The steep slope of the binding isotherm illustrates strong adsorption to GCN4-pII constructs at low LPS concentrations, with estimated apparent dissociation constants (K_D,app_) in the picomolar range (**Figure 5**). This is in concordance with the high affinity observed in the SPR experiments. Interestingly, the affinity of GCN4-pII, with a K_D,app_ in the picomolar range, is 3-5 orders of magnitude higher than the human LPS immune receptors TLR4 (141 µM), CD14 (74 nM), MD-2 (2.33 µM), and LPS binding protein (3.5 nM) (Viriyakosol et al., 2000; Shin et al., 2007; Basauri et al., 2020). The dissociation constants obtained with GCN4-pII are also 1-6 orders of magnitude higher than for the antibiotic polymyxin B (48 µM) (Thomas and Surolia, 1999), and even higher than that of peptide affibodies specifically designed with the aim of highest achievable affinity (Matsumoto et al., 2010).

### 3.3 Comparing GCN4-pII to the LAL assay as an LPS detection assay

**Figure 6:**
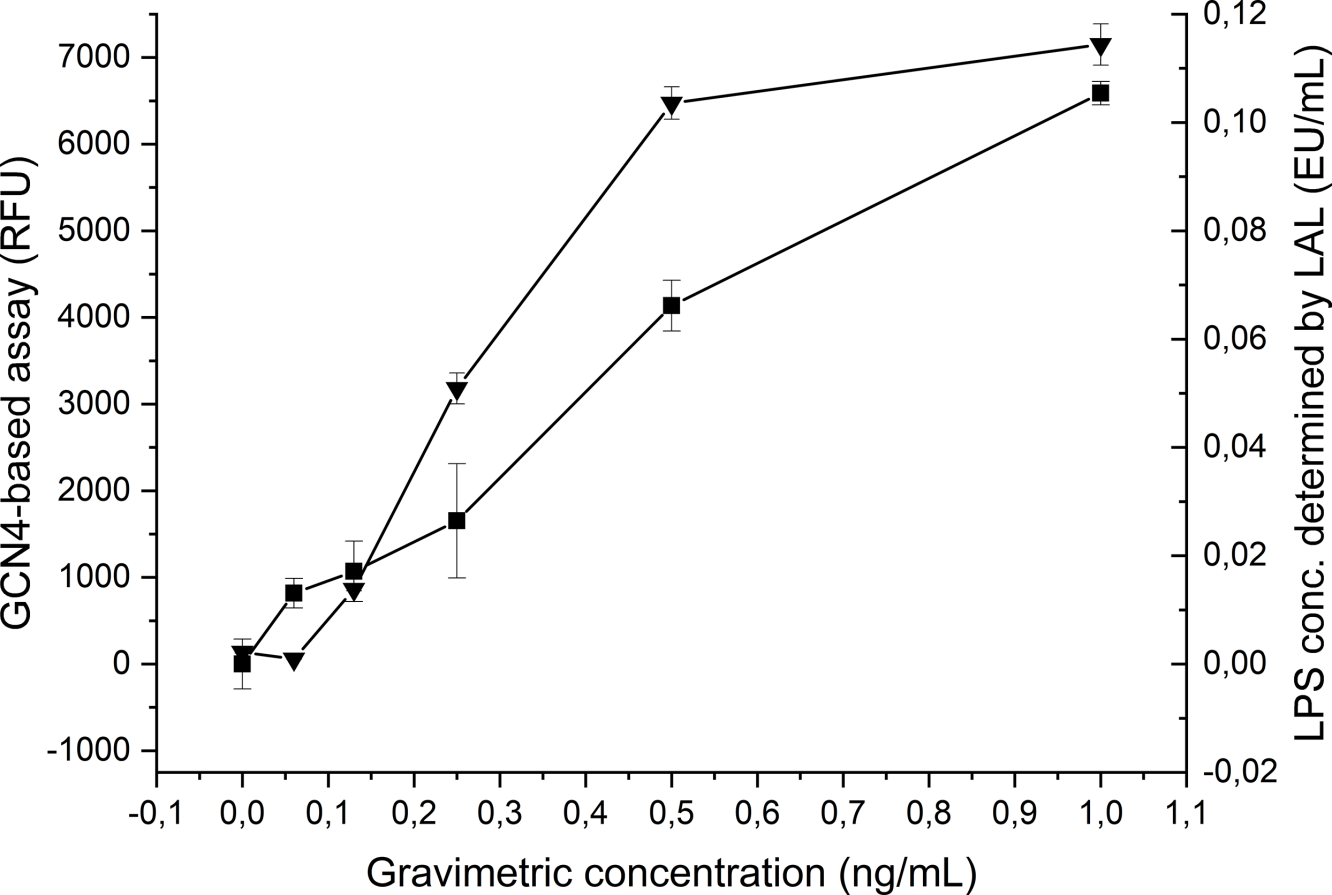
Comparison of the GCN4-pII based (_▀_) and LAL based (▲) LPS detection sensitivity. The horizontal axis denotes the LPS concentration. The left vertical axis denotes the fluorescence signal for GCN4-pII based detection, and the right axis denotes the LPS concentration determined by LAL.

Our results demonstrated that GCN4-pII binds to the conserved lipid A moiety of LPS with a high affinity, binding to LPS from a broad range of Gram-negative species. We therefore wanted to investigate whether GCN4-pII could be a suitable candidate as the basis of a LPS detection assay. We modified our ELITA-assay setup by using biotinylated *E. coli* LPS in lieu of *S.* typhimurium LPS, which could be detected directly using HRP-conjugated streptactin. This configuration removed several steps from the original protocol and increased the sensitivity dramatically. The sensitivity of the GCN4-pII based LPS detection was compared to the current leading LPS-detection solution, the LAL-assay, by measuring each sample dilution in parallel using LAL. The GCN4-pII based assay could detect LPS at concentrations as low as 0.062 ng/mL, while the LAL-assay could detect with a sensitivity as low as 0.125 ng/mL, demonstrating that the GCN4-pII in principle can detect LPS with a similar or higher sensitivity than the LAL assay (**Figure 6**).

### 3.4 GCN4-pII dissolves LPS micelles

We noticed during our work that adding GCN4-pII-containing constructs to LPS caused a visible reduction of turbidity, indicating that the LPS micelles are disrupted and broken down. To investigate the effect of GCN4-pII on LPS in solution, we used a synthetic peptide comprising only the GCN4-pII sequence. Prior to further experiments, we confirmed that the peptide exists in a homogenous state in solution, using NMR spectroscopy (**Figure S7A**). A LAL masking assay shows at least 89% neutralizing effect on LPS at a GCN4-pII concentration of 1 nM, confirming that the peptide alone binds LPS and prevents it from activating Factor C (Schwarz et al., 2017) (**Figure S7B**). Comparison of CD spectra between GCN4-pII alone and in complex with LPS confirms that the peptide retains its secondary structure comprised of α-helices upon binding (**Figure S7C**). Together, these data confirm that the synthetic GCN4-pII peptide binds LPS, and that the binding observed with the SadA constructs is not caused by a motif that is specific to *Salmonella* SadA.

**Figure 7:**
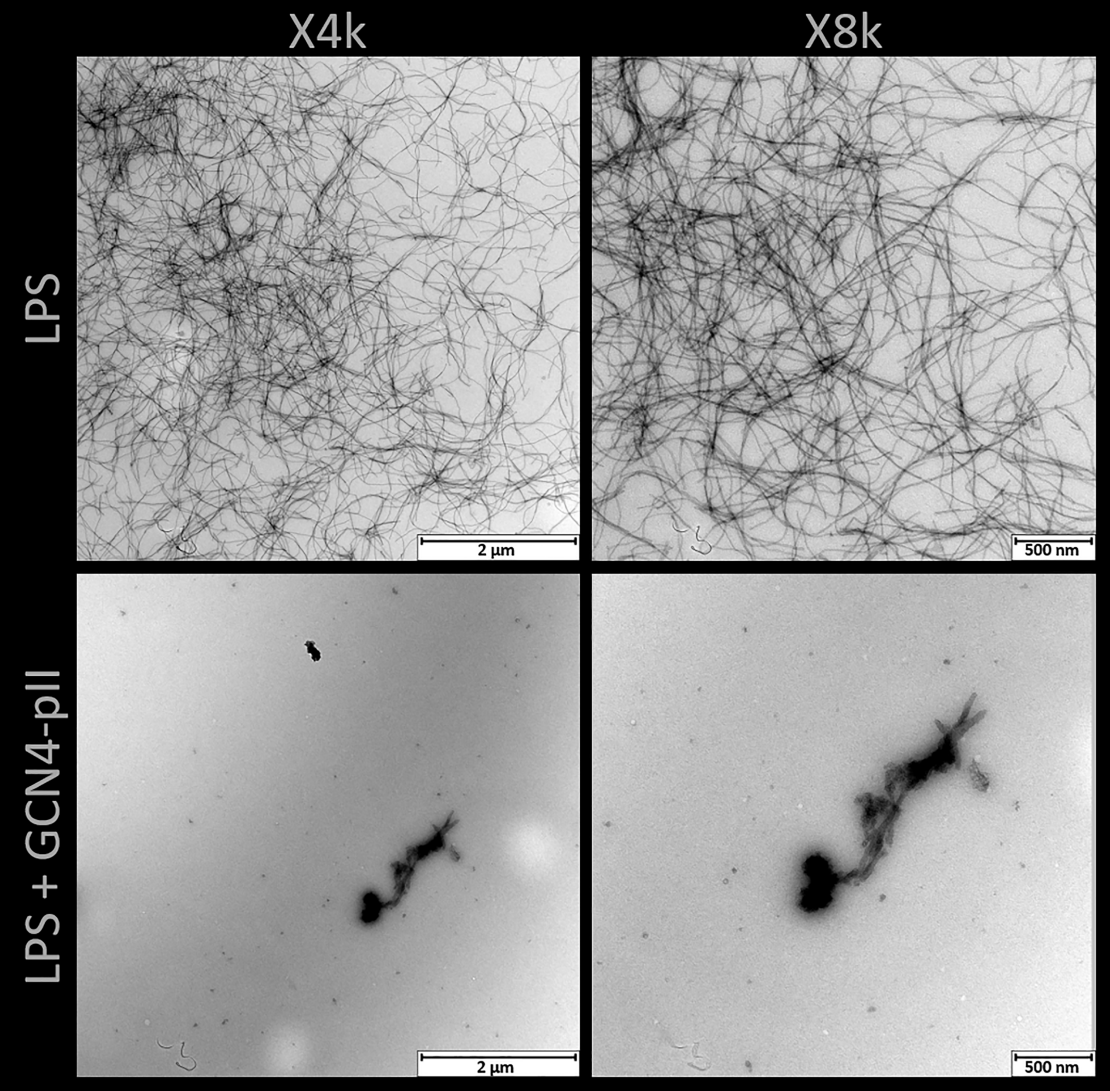
TEM of rough LPS from *S.* typhimurium with and without GCN4-pII present in a 1:4 stoichiometric ratio. LPS alone forms worm-like micelles with lengths up to several hundred nanometers. Addition of GCN4-pII breaks down the worm-like structure, leaving occasional fragmented micelles.

**Figure 8:**
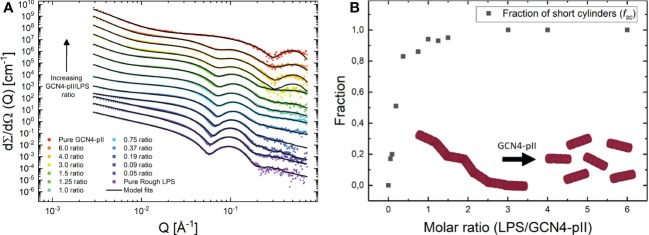
**(A)** SAXS data from samples containing mixes of rough LPS and GCN4-pII in varying molar ratios (GCN4-pII/LPS). For clarity, the data has been shifted vertically with factors 6*^n^* where *n* goes from 0 to 12 from bottom to top. The solid lines display fits of the quantitative scattering models. **(B)** Fraction of short cylinders as a function of molar ratio (GCN4-pII/LPS) obtained from the fits shown to the left.

The effects of GCN4-pII on the structure of LPS micelles were studied using two independent methods, TEM and SAXS (**Figures 7**, **8**). Rough LPS observed with TEM forms cylindrical micelles with a total diameter of around 10 nm and lengths ranging up to hundreds of nm (**Figure 7**), in line with earlier reports using cryo-EM (Richter et al., 2011; Broeker et al., 2018). Following incubation with equimolar amounts of GCN4-pII, the micellar structures completely disappear, leaving occasional small fragmented micelles that we interpret as a result of peptide-LPS co-assemblies. The determination of structural transitions was analyzed using SAXS, which combined with modelling, provided detailed structural information of the binding partners (see Materials and methods section and SI for more details). The experimental scattering curves from samples containing either LPS alone or GCN4-pII/LPS mixtures of varying molar ratios can be seen in **Figure 8A** together with the corresponding model fits. For pure LPS, the scattering at low *Q* follows a decay of *~Q* ^-1.7^, being indicative of worm-like structures (Arleth et al., 2002). However, when the molar ratio is increased, the scattering at low to medium *Q* rather exhibit a plateau followed by a decay closer to *Q* ^-1^, which is typical of short cylindrical micelles (Pedersen, 2008; Jensen et al., 2014). It should also be noted that the steep decay at the lowest *Q*-values (~Q^-4^) visible at high molar ratios can likely be attributed to large aggregates of presumably LPS and GCN4-pII, and this was consequently accounted for in the modelling. The fits of the scattering data confirm the EM observations and the qualitative analysis of the scattering curves, as the sample containing pure LPS was well described by the worm-like micelle model using a diameter of around 11 nm. While we could quantify the flexibility of the worms, the contour length of the worms could not be determined by SAXS due to the limited *Q*-range and resulting maximal resolvable length around 100 nm. Moreover, the scattering from the samples containing LPS and GCN4-pII in various molar ratios were fitted using the described scattering model with varying fractions of worm-like LPS micelles to short cylinders (broken-down worms). This fraction was plotted as a function of the GCN4-pII/LPS ratio (**Figure 8B**). The addition of GCN4-pII at low ratios disturb the worm-like micelles, and when approaching a 1:1 ratio, the short cylinders become the predominant species, which is in concordance with the EM data. To summarize, EM and SAXS clearly show that GCN4-pII readily disturbs, and breaks down LPS micelles in solution, however, the exact mechanism of this effect has yet to be elucidated in full detail and requires further investigations.

## 4 Discussion

We originally set out to study a putative interaction between trimeric SadA domains and LPS. Our results however, show that the GCN4-pII adapters used to stabilize our constructs display an extremely high affinity for the lipid A moiety of LPS. The only effective buffer for regenerating the SPR chip was composed of a mixture of detergents, and GCN4-pII readily dissolves LPS micelles in solution. This strongly suggests that that the interaction between GCN4-pII and LPS is predominantly of a hydrophobic character, meaning that the GCN4-pII interacts to large degree with the acyl tails of lipid A. We were unable to find any other examples of an LPS-binding trimeric coiled-coil motif in literature, meaning that the binding mechanism is currently unknown. However, crystal structures of fusion proteins containing GCN4-pII reported earlier (Hartmann et al., 2012) show that the γ_2_ and δ-carbons belonging to the core isoleucins protrude from the core to some extent (**Figure 1B**), forming hydrophobic surfaces along the coiled-coil grooves. It is conceivable that one or more of the lipid A acyl tails can align along these grooves to form the hydrophobic interactions, a model that also explains how GCN4-pII can break down LPS micelles (Lee, 2003). However, GCN4-pII also has a C-terminal patch of cationic residues, and our experiments cannot rule out that these also contribute to the interaction by coordinating one or both of the phosphate groups belonging to lipid A. We noticed that the GCN4-pII sequence shares sequence features with cationic α-helical antimicrobial peptides (α-AMPs), a class of AMPs known to bind and neutralize LPS, albeit with a 2-3 orders of magnitude lower affinity (Scott et al., 2011; Sun and Shang, 2015). α-AMPs are characterized by an α-helical structure with branched hydrophobic residues often appearing in the heptad a and d positions accompanied by a patch of cationic residues at either terminus (Doi et al., 2000; Liang et al., 2006; Wang et al., 2016; Sinha et al., 2017; Simpson and Trent, 2019). Indeed, their mode of binding to LPS is composed of a combination of hydrophobic and electrostatic forces, and several α-AMPs disrupt LPS micelles (Wang, 2008; Sun and Shang, 2015). However, α-AMPs do form trimeric coiled-coil complexes in solution, but usually assemble into pore-forming elements in the outer membrane of Gram-negative bacteria (Doi et al., 2000). We conducted a viability assay on *E. coli* and *Salmonella* bacteria that shows that GCN4-pII does not have any observable antimicrobial properties (not shown). This indicates that, although of GCN4-pII-LPS interactions share some structural characteristics with those of AMPs, there must still be some fundamental differences in function. Interestingly, it was recently shown that leucine substitutions in the heptad repeats of the AMP piscidin-1 induce dimeric coiled-coil formation, and reduce cytotoxicity while retaining LPS-neutralizing activity (Kumar et al., 2016). More work is needed to elucidate the GCN4-pII/LPS interaction on the molecular level, also to answer whether this is an interaction specific to GCN4-pII only, or can be generalized for other trimeric coiled-coil motifs.

The GCN4-pII adaptor has widely been used in fusion proteins to stabilize trimeric complexes for more than two decades and, to our knowledge, the strong interaction with LPS has remained unknown until now. A majority of the protocols we have checked use purification strategies where constructs are expressed as inclusion bodies and/or are purified under denaturing conditions, which probably prevents unintended co-purification of LPS from the expression host, or used non-bacterial expression hosts (Yang et al., 2002; Alvarez et al., 2008; Cornelissen et al., 2010; Hartmann et al., 2012; Deiss et al., 2014). LPS is not stained by Coomassie (Marolda et al., 2006), and is normally not visible in SDS-PAGE gels, which could explain why this interaction has gone unnoticed for so long. This work should serve as a warning to researchers using GCN4-pII to stabilize their trimeric complexes, particularly for those who use it for immunological or cell biology work, where an LPS contamination can seriously compromise the experiments.

We demonstrated that GCN4-pII binds to LPS isolated from a broad range of Gram-negative species. We postulated that the high binding affinity combined with the broadness of binding and the fact that GCN4-pII binds to the conserved lipid A moiety of LPS, could make it a suitable candidate for a novel, synthetic LPS detection assay. We demonstrated as a proof-of-principle that a GCN4-pII based assay detects LPS with similar or higher sensitivity than the industry gold standard for LPS detection, the LAL-assay. The LAL-assay relies on blood clotting factors extracted from the blood of horseshoe crabs (Tamura et al., 2021), making it not sustainable as these crabs are an endangered species. Increasing demand from the pharmaceutical industry still drives a global overharvest of horseshoe crabs, necessitating novel detection solutions, and we hope that our findings can provide future solutions. However, our proof-of-concept assay relied on biotinylated LPS, and further research and development are necessary to translate our findings into a generally applicable detection method.

## Data availability statement

The original contributions presented in the study are included in the article/**Supplementary Material**. Further inquiries can be directed to the corresponding author.

## Author contributions

DH and DL conceived the project. DH expressed and purified the proteins used, purified LPS, performed ELITA assays with data analysis, SPR experiments with data analysis, and SAXS data collection, and prepared the paper with input from all authors. NB purified LPS and provided reagents for the study. SB analyzed SPR and ELITA data. MC and RL analyzed and modelled SAXS data. PK performed data collection of NMR and CD data. SB and DL supervised the project. All authors contributed to the article and approved the submitted version.
