# Supplementary material for: A trimeric coiled-coil motif binds bacterial lipopolysaccharides with picomolar affinity
Source: Front Cell Infect Microbiol. 2023 Feb 16;13:1125482. doi: 10.3389/fcimb.2023.1125482 (PMC9978483; doi:10.3389/fcimb.2023.1125482)
Supplement: Supplementary file 1 [file DataSheet_1.pdf]

Supplementary Material

A trimeric coiled-coil motif binds bacterial lipopolysaccharides with picomolar affinity

Daniel Hatlem, Mikkel Christensen, Nina K. Broeker, Per E. Kristiansen, Reidar Lund, Stefanie Barbirz, Dirk Linke*

*** Correspondence:** Dirk Linke: dirk.linke@ibv.uio.no

1 Supplementary Tables

Supplementary Table 1 *Bacterial* strains used for LPS production. *S.* typhimurium refers to Salmonella enterica ssp. enterica serovar Typhimurium.

Strain	Type	Notes
Bartonella henselae	Smooth	*B. henselae* LPS has a significantly reduced endotoxic potential compared to other LPS types. The lipid A moiety is penta-acylated, containing a significantly longer (28:0(27-OH)) fatty acid (Zähringer et al., 2004).
Neisseria lactamica	Rough	LPS from *Neisseria* spp. are commonly referred to as lipooligosaccharides (LOS). The lipid A structure is similar to that of *S.* typhimurium, but with the addition of a phosphoethanolamine modification to the distal phosphoglucosamine group. LOS lack the O-antigen commonly found in other Gram-negative species, and the core-oligo saccharides are larger and branched (Kahler et al., 2018).
Porphyromonas gingivalis	Smooth	Acquired from commercial vendor (InvivoGen). *P. gingivalis* lipid A is penta-acylated, with longer and branched fatty acids (Nativel et al., 2017).
***S.* typhimurium**	Smooth	
***S.* typhimurium ΔwaaC**	Deep rough	Deletion of heptosyltransferase WaaC, responsible for transfer of heptose to the Kdo-moiety of LPS-precursor (Sirisenas et al., 1992).
***S.* typhimurium ΔwaaL**	Rough	Deletion of WaaL ligase, responsible for ligating O-antigen to core-oligo saccharide (Raetz and Whitfield, 2002).
Vibrio cholerae	Smooth	The lipid A moiety of *V. cholera* differs from *S.* typhimurium lipid A by an additional phosphoethanolamine modification on the proximal phosphoglucosamine, and secondary fatty acids bound to the amide-linked fatty acids in lieu of the O-linked fatty acids. The core oligosaccharide and O-antigen are smaller than their counterparts found in *S.* typhimurium LPS (Chatterjee and Chaudhuri, 2003).

Supplementary Table 2 Fit parameters obtained from fitting the full model to scattering data obtained from samples of LPS and GCN4-pII with varying molar ratios (GCN4-pII/LPS). Lengths above $\sim\mathbf{1000}$ Å are not resolvable due to the limited $\mathbf{Q}$-range. * denotes fixed parameters.

$c_{pep.}$ [mg/mL]*	c_{LPS} [mg/mL]*	Molar ratio	R_{core} [Å]	t_{shell} [Å]	R_{tot} [Å]	ϵ	f_{sc}	f_{bp}	L_{core} [Å]	L_{worm} [Å]*	b [Å]	d_{tail} [g/cm^3]	d_{head} [g/cm^3]*
0	0.75	0	22.7 ± 0.9	32.4 ± 2.3	55.1 ± 3.2	0.69 ± 0.04	0	N/A	N/A	> 1000	131 ± 31	0.86 ± 0.02	1.67 ± 0.05
0.038	0.75	0.05	23.6 ± 0.9	29.7 ± 2.1	53.3 ± 3.1	0.74 ± 0.04	0.17	1.0	70 ± 8	> 1000	173 ± 41	0.87 ± 0.02	1.67 ± 0.05
0.077	0.75	0.09	23.7 ± 0.9	27.8 ± 2.0	51.5 ± 2.9	0.74 ± 0.04	0.20	1.0	50 ± 6	> 1000	400 ± 95	0.89 ± 0.02	1.67 ± 0.05
0.15	0.75	0.19	23.7 ± 0.9	26.0 ± 1.9	49.7 ± 2.8	0.72 ± 0.04	0.51	1.0	50 ± 6	> 1000	> 1000	0.91 ± 0.02	1.67 ± 0.05
0.31	0.75	0.37	23.7 ± 0.9	23.6 ± 1.7	47.3 ± 2.6	0.73 ± 0.04	0.83	1.0	45 ± 5	> 1000	> 1000	0.91 ± 0.02	1.67 ± 0.05
0.61	0.75	0.75	21.9 ± 0.9	24.1 ± 1.7	46.0 ± 2.6	0.72 ± 0.04	0.84	0.88	50 ± 6	> 1000	> 1000	0.91 ± 0.02	1.67 ± 0.05
0.82	0.75	1.0	21.6 ± 0.9	25.0 ± 1.8	46.6 ± 2.7	0.72 ± 0.04	0.90	0.70	53 ± 6	> 1000	> 1000	0.91 ± 0.02	1.67 ± 0.05
1.02	0.75	1.25	21.2 ± 0.8	24.0 ± 1.7	45.2 ± 2.5	0.72 ± 0.04	0.90	0.68	50 ± 6	> 1000	> 1000	0.91 ± 0.02	1.67 ± 0.05
1.23	0.75	1.5	22.0 ± 0.9	24.3 ± 1.7	46.3 ± 2.6	0.70 ± 0.04	0.92	0.55	52 ± 6	> 1000	> 1000	0.90 ± 0.02	1.67 ± 0.05
1.5	0.46	3.0	21.9 ± 0.9	24.0 ± 1.7	45.9 ± 2.6	0.70 ± 0.04	0.92	0.20	50 ± 6	> 1000	> 1000	0.89 ± 0.02	1.67 ± 0.05
1.5	0.34	4.0	21.9 ± 0.9	24.0 ± 1.7	45.9 ± 2.6	0.67 ± 0.03	0.92	0.20	50 ± 6	> 1000	> 1000	0.89 ± 0.02	1.67 ± 0.05
1.5	0.23	6.0	21.8 ± 0.9	23.9 ± 1.7	45.7 ± 2.6	0.65 ± 0.03	0.94	0.20	50 ± 6	> 1000	> 1000	0.87 ± 0.02	1.67 ± 0.05

Supplementary Table 3 Fit parameters obtained from fitting the trimeric peptide bundle model to scattering data obtained from a sample of pure GCN4-pII. The obtained values were used as a basis for the fitting of the LPS/peptide mixtures. * denotes fixed parameters.

c_{pep} [mg/mL]*	R [Å]	L [Å]	f	C	ξ [Å]	d_{pep} [g/cm^3]
1.22	2.36	133	2.66	6050	686.7	1.35

Supplementary Table 4 Kinetic and statistical parameters of the ELITA-assay determined by non-linear regression using the Hill equation.

	**K9**	**K14**
**Y_{max}**	0.70501 ± 0.01227 Abs/nM	0.61075 ± 0.02428 Abs/nM
**$K_{0.5}$**	0.07253 ± 0.00596 nM	0.06356 ± 0.00986 nM
**n**	0.69342 ± 0.02735	0.66018 ± 0.03779
**R^2**	0.998	0.996

2 Supplementary Figures

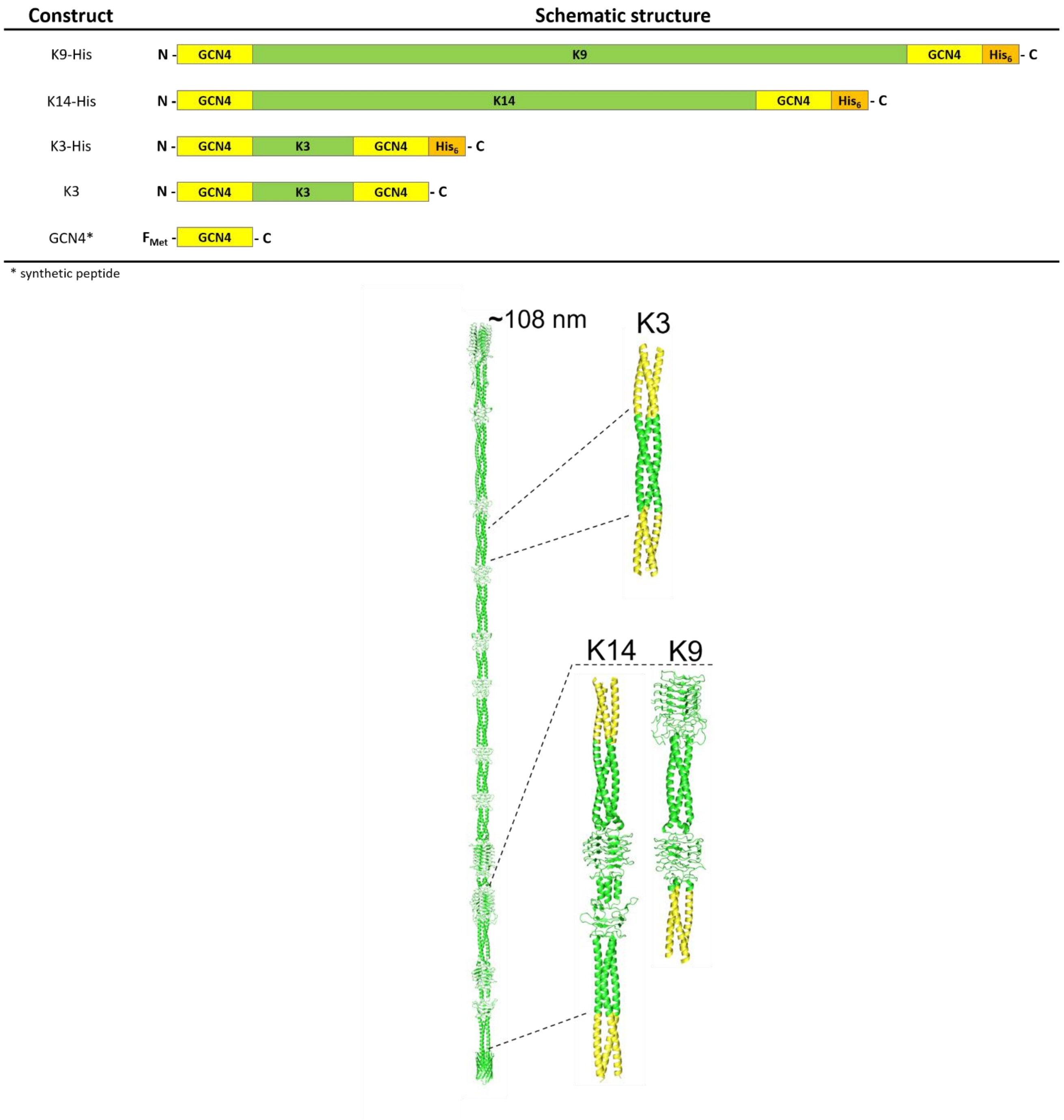

Supplementary Figure 1 Top: Schematic overview of the constructs and peptide used in this study. Bottom: model of the full length *Salmonella* adhesin A (SadA), and the constructs used in this study (PDB-ID: 2YO1, 2YO3, and 2WPQ) (Hartmann et al., 2012). The sequence originating from *Salmonella* adhesin A (SadA) is colored green, and the GCN4-pII fusion segments used to stabilize the constructs are colored yellow.

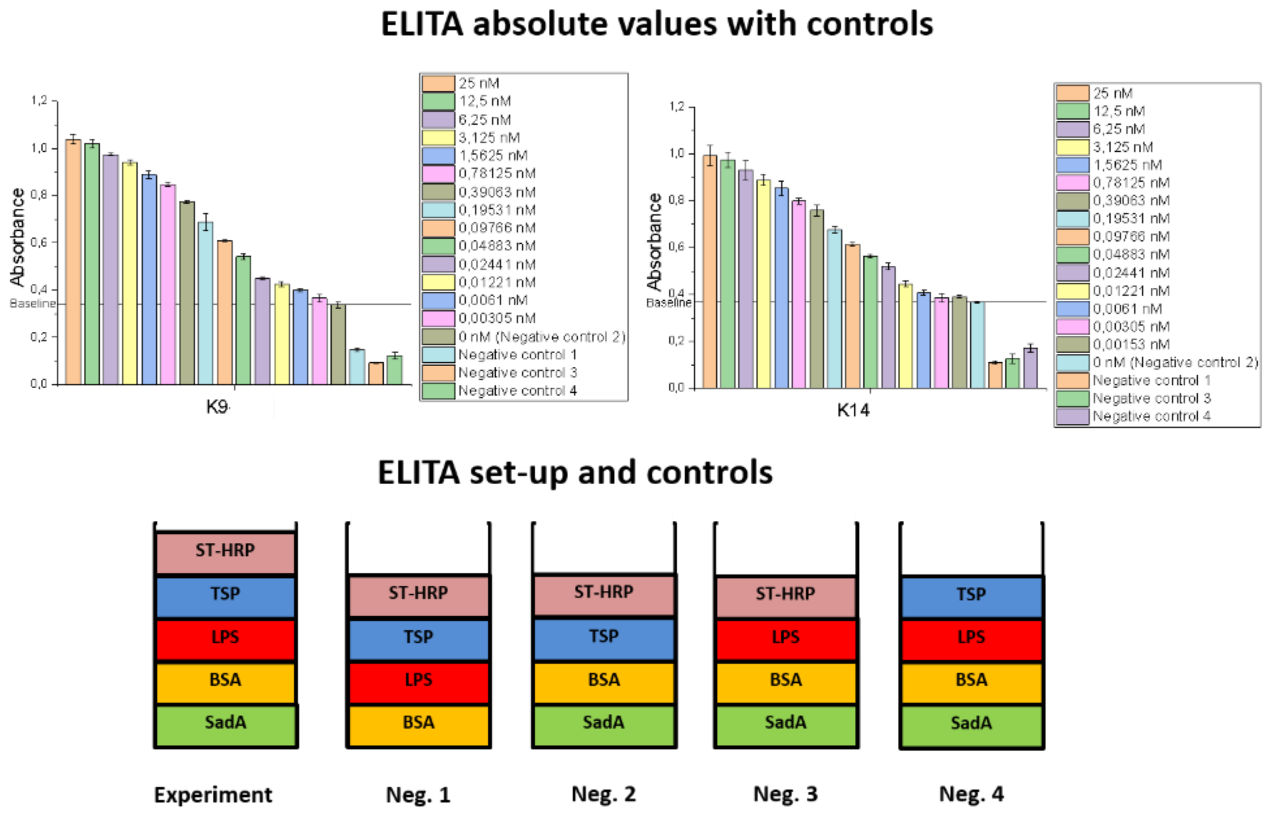

Supplementary Figure 2 Set up and absolute values of the ELITA experiments. SadA = Salmonella component K9 or K14. BSA = Bovine serum albumin. TSP = phage tailspike protein. ST-HRP = streptactin conjugated horseradish peroxidase.

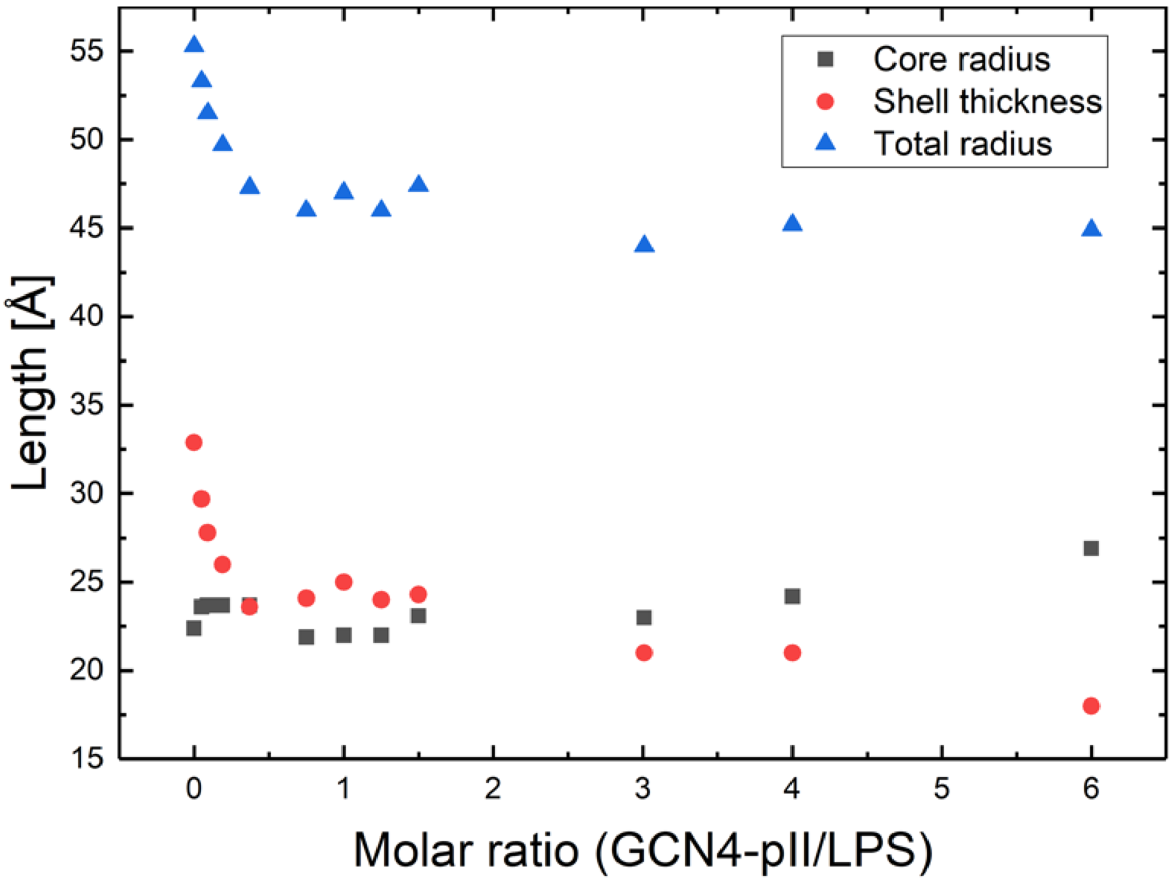

Supplementary Figure 3 Cross-section dimensions of the LPS micelles as a function of molar ratio (GCN4-pII/LPS) obtained from model fitting of SAXS curves.

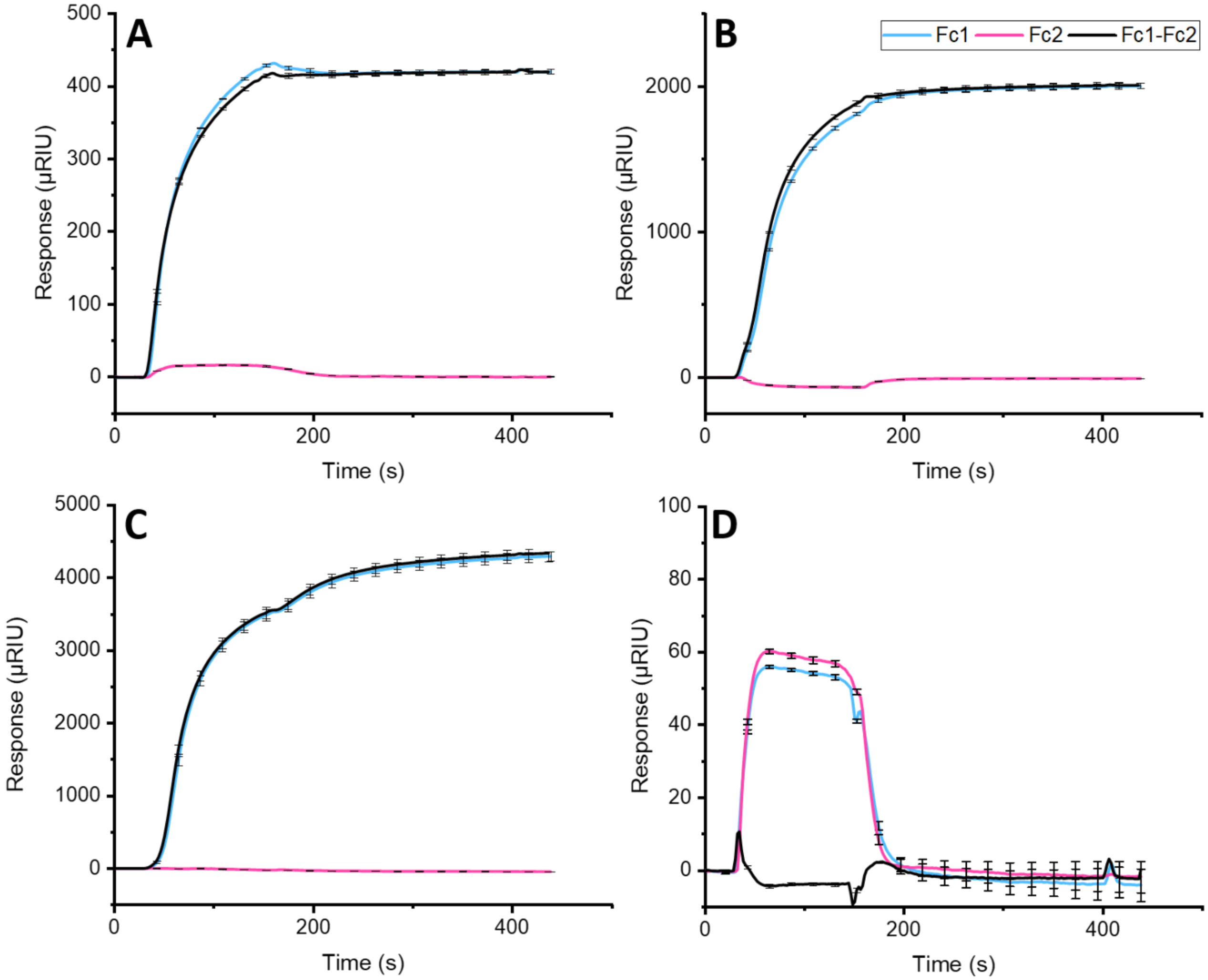

Supplementary Figure 4 SPR Flow channel 1 (Fc1), Flow channel 2 (Fc2), and difference Fc1-F2 curves for immobilized SadA K9 with different S. typhimurium LPS. (**A**) Smooth LPS (**B**) Rough LPS (**C**) Deep rough LPS (**D**) Polysaccharide derived from LPS.

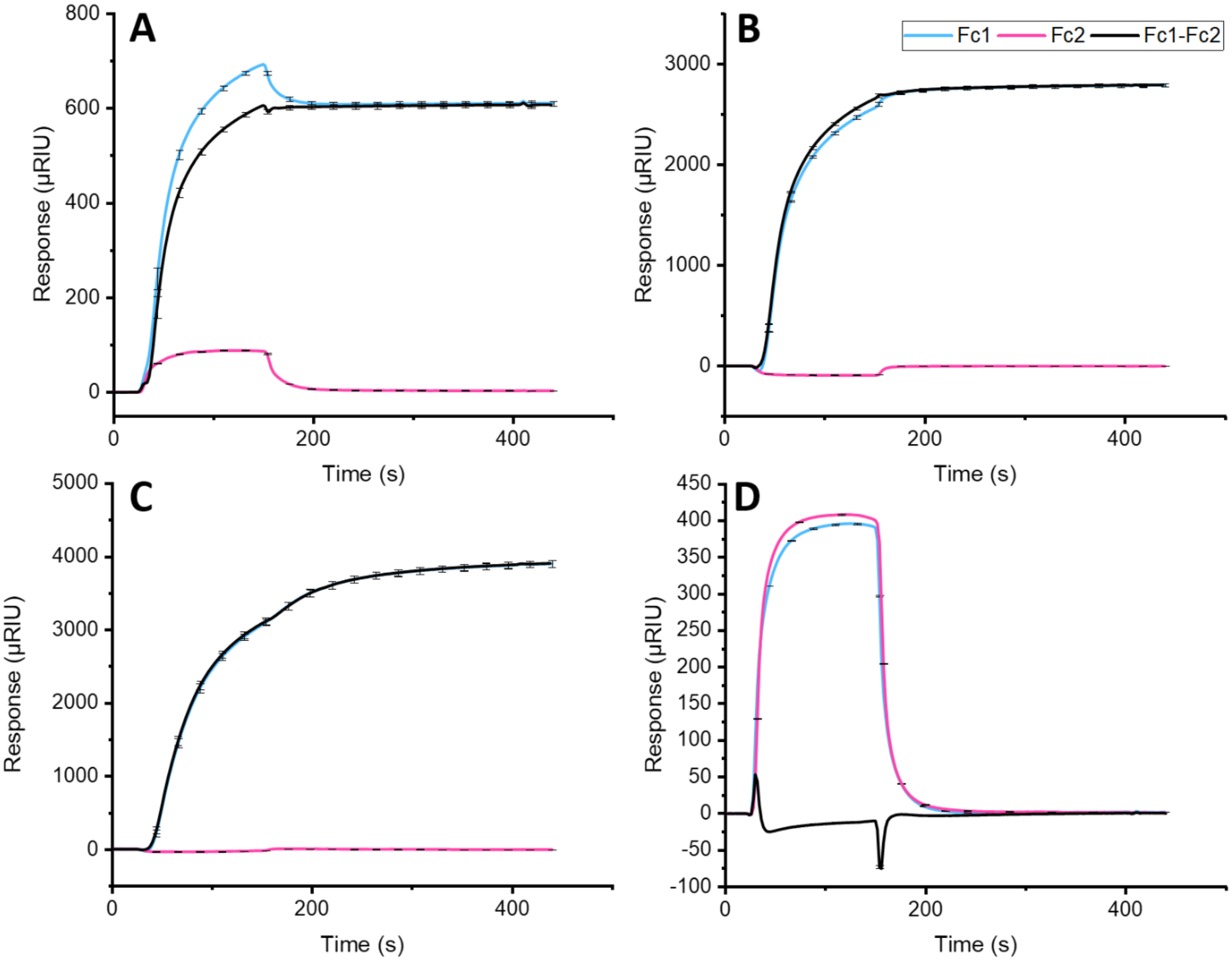

Supplementary Figure 5 SPR Flow channel 1 (Fc1), Flow channel 2 (Fc2), and difference Fc1-F2 curves for immobilized SadA K14 with different S. typhimurium LPS. (**A**) Smooth LPS (**B**) Rough LPS (**C**) Deep rough LPS (**D**) Polysaccharide derived from LPS. Signal normalized to K9.

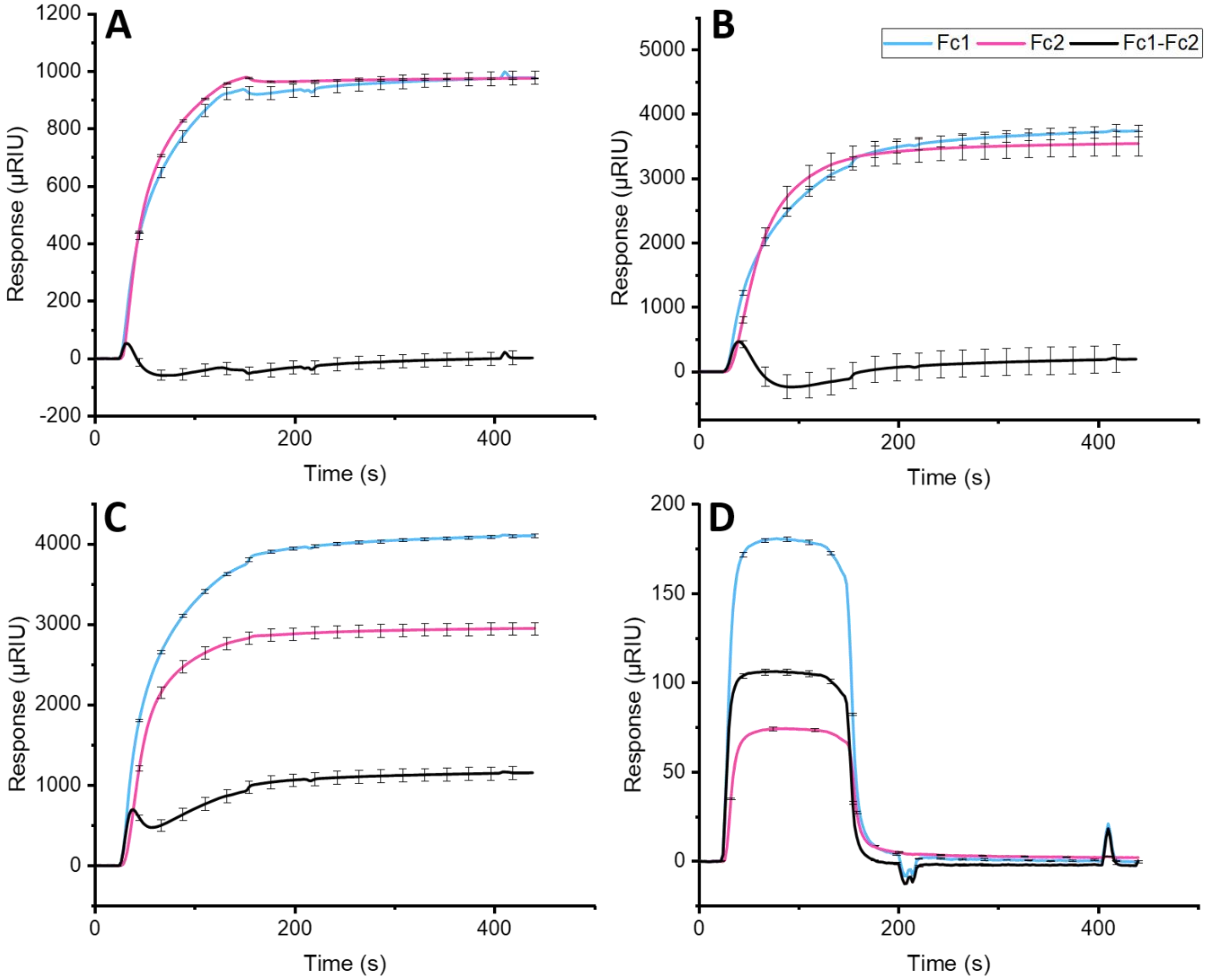

Supplementary Figure 6 SPR Flow channel 1 (Fc1), Flow channel 2 (Fc2), and difference Fc1-F2 comparison curves for immobilized SadA K3 (Fc1 channel) and SadA K3-His (Fc2 channel). This setup differs from the experiments shown in **Figure 4** and **5** by coupling protein to both the measurement and reference channel for comparison of protein with- and without a His-tag. (**A**) Smooth LPS (**B**) Rough LPS (**C**) Deep rough LPS (**D**) Polysaccharide derived from LPS. Signal normalized to K9.

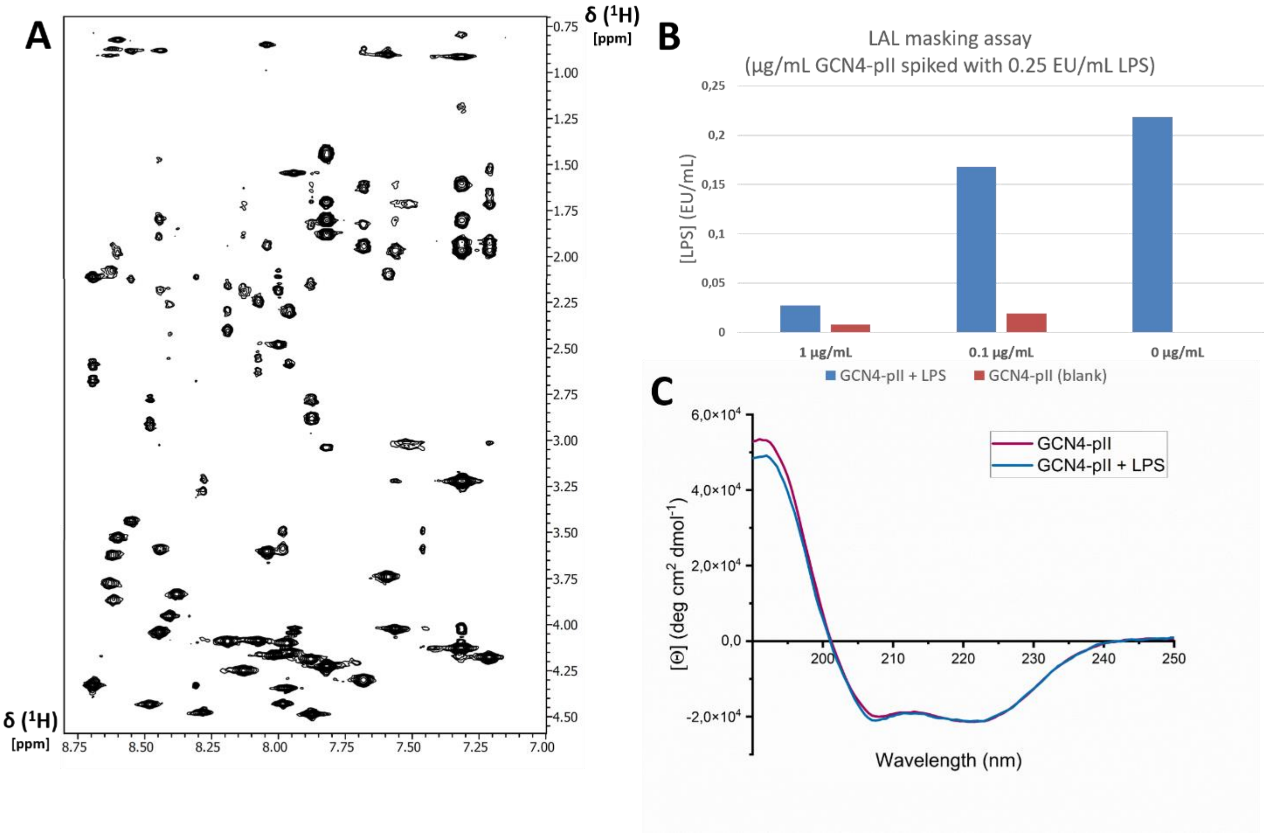

Supplementary Figure 7 (**A**) Fingerprint region of a ^{1}H-^{1}H-TOCSY spectrum of GCN4-pII. All 29 expected spin systems where well resolved and assignable without indications of peak splitting, indicating that the peptide exist in a homogenous state in solution. (**B**) LAL assay demonstrating the masking effect of 0.1 and 1 µg/mL GCN4-pII on 0.25 EU/mL E. coli LPS standard demonstrating that the synthetic GCN4-pII peptide binds LPS in solution. (**C**) CD spectra of GCN4-pII alone and in presence of LPS demonstrating little variation in secondary structure composition before and after binding to LPS.

3 References

Chatterjee, S. N., and Chaudhuri, K. (2003). Lipopolysaccharides of Vibrio cholerae: I. Physical and chemical characterization. *Biochim. Biophys. Acta - Mol. Basis Dis.* 1639, 65–79. doi: 10.1016/J.BBADIS.2003.08.004.

Hartmann, M. D., Grin, I., Dunin-Horkawicz, S., Deiss, S., Linke, D., Lupas, A. N., et al. (2012). Complete fiber structures of complex trimeric autotransporter adhesins conserved in enterobacteria. *Proc Natl Acad Sci U S A* 109, 20907–20912. doi: 10.1073/pnas.1211872110.

Kahler, C. M., Nawrocki, K. L., Anandan, A., Vrielink, A., and Shafer, W. M. (2018). Structure-Function Relationships of the Neisserial EptA Enzyme Responsible for Phosphoethanolamine Decoration of Lipid A: Rationale for Drug Targeting. *Front. Microbiol.* 9, 1922. doi: 10.3389/fmicb.2018.01922.

Nativel, B., Couret, D., Giraud, P., Meilhac, O., D'Hellencourt, C. L., Viranaïcken, W., et al. (2017). Porphyromonas gingivalis lipopolysaccharides act exclusively through TLR4 with a resilience between mouse and human. *Sci. Rep.* 7, 1–12. doi: 10.1038/s41598-017-16190-y.

Raetz, C. R. H., and Whitfield, C. (2002). Lipopolysaccharide Endotoxins. *Annu. Rev. Biochem.* 71, 635–700. doi: 10.1146/annurev.biochem.71.110601.135414.

Sirisenas, D. M., Brozekj, K. A., Maclachlansli, P. R., Sanderson$, K. E., and Raetzqii, C. R. H. (1992). The rfaC gene of Salmonella typhimurium. Cloning, sequencing, and enzymatic function in heptose transfer to lipopolysaccharide.

Zähringer, U., Lindner, B., Knirel, Y. A., Van Den Akker, W. M. R., Hiestand, R., Heine, H., et al. (2004). Structure and biological activity of the short-chain lipopolysaccharide from Bartonella henselae ATCC 49882T. *J. Biol. Chem.* 279, 21046–21054. doi: 10.1074/jbc.M313370200.
